# 3D‐Printed Metal‐Supported MOF‐Heteropoly Acid Nanozyme for High‐Performance Peroxidase‐Mimic Activity and Ultra‐Sensitive Glucose Detection

**DOI:** 10.1002/advs.202524355

**Published:** 2026-03-12

**Authors:** Paramita Koley, Ranjithkumar Jakku, Subhash Chandra Shit, Jang Mee Lee, Guy N. L. Jameson, Tayebeh Hosseinnejad, Selvakannan Periasamy, Deshetti Jampaiah, Amrit Raj Paul, Ylias Sabri, Suresh K. Bhargava

**Affiliations:** ^1^ Centre for Advanced Materials & Industrial Chemistry (CAMIC) School of Applied Sciences RMIT University Melbourne Australia; ^2^ Department of Energy Engineering/KENTECH Institute for Environmental and Climate Technology Korea Institute of Energy Technology (KENTECH) Naju Republic of Korea; ^3^ School of Chemistry and Bio21 Molecular Science and Biotechnology Institute The University of Melbourne Parkville Victoria Australia

**Keywords:** 3D printed catalysts, glucose detection, in situ electron paramagnetic resonance spectroscopy, metal organic frameworks‐polyoxometallate, X‐ray absorption spectroscopy

## Abstract

The development of nanozymes combining high catalytic activity, mechanical robustness, and scalable fabrication is crucial for next‐generation biomedical sensing. However, most current 3D‐printed diagnostic platforms rely on polymeric substrates that suffer from limited reusability, weak mechanical strength, and poor long‐term stability. Here, we report a sustainable and robust nanozyme system based on a 3D‐printed Ti–Al─V alloy substrate, chosen for its excellent mechanical integrity, reusability, and intrinsically rough surface that promotes metal–organic framework growth. For the first time, an iron‐based MOF (Fe‐BTC) is directly grown on a 3D‐printed Ti─Al─V substrates with in situ incorporation of phosphomolybdic acid, forming a highly active Fe‐BTC‐PMA nanozyme. The rough metallic substrates enable uniform MOF nucleation and strong interfacial anchoring, while electronic interactions between the Ti─Al─V substrate and the Fe‐BTC‐PMA framework facilitate efficient charge transfer and accelerated redox kinetics. Spectroscopic analyses, including XANES, EXAFS, and XPS, reveal PMA‐induced modulation of the iron coordination environment and charge redistribution. These results are supported by kinetic studies, in situ electron paramagnetic resonance spectroscopy, and density functional theory calculations. Compared with conventional powder nanozymes, the integrated platform exhibits enhanced catalytic activity, superior stability, and excellent reusability, enabling sensitive and reliable glucose sensing.

## Graphical abstract

A hierarchically structured Fe‐BTC‐PMA immobilized 3D‐printed platform is engineered as a robust artificial enzyme for colorimetric glucose sensing. The hybrid architecture efficiently activates H_2_O_2_ to drive TMB oxidation. This integrated system enables sensitive, reusable, and scalable detection with enhanced catalytic stability and analytical reliability.

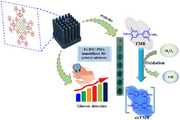

## 1 Introduction

Metal‐organic frameworks (MOFs) are porous crystalline materials formed through the self‐assembly of polydentate organic ligands and metal nodes [1, 2, 3]. Their distinctive microstructures provide them with attractive properties, including high specific surface areas, tunable porosity, and well‐defined cavities, which have enabled widespread application in small‐molecule sensing, gas storage, and catalysis [4, 5]. In particular, substantial progress has been made in exploiting MOFs as enzyme mimics [6, 7, 8]. For instance, Zhou and co‐workers reported that the Fe‐based porphyrinic MOF PCN‐222, constructed from tetrakis(4‐carboxyphenyl) porphyrin(TCPP) ligands, exhibited pronounced peroxidase‐like activity toward the oxidation of various substrates, whereas analogous frameworks incorporating manganese, cobalt, nickel, copper, or zinc centers displayed markedly lower catalytic performance [9]. Ma and colleagues subsequently demonstrated a highly stable mesoporous metal–metalloporphyrin framework(MMPF‐6), assembled from iron‐based ligands and Zr_6_O_8_(CO_2_)_8_(H_2_O)_8_ secondary building units, which also showed notable peroxidase mimic behavior [10]. Among MOFs, iron‐based systems such as MIL‐53(Fe) [11], Fe‐NH_2_‐MIL‐88 [12], MIL‐68(Fe) [13], and MIL‐100(Fe), [14] have attracted particular attention as nanozymes because of their ability to catalytically decompose hydrogen peroxide to generate reactive oxygen species for bioanalytical applications [15, 16]. Nevertheless, most reported MOF nanozymes are 3D bulk crystals. Their inherently narrow pore apertures can inhibit substrate diffusion, thereby limiting access to active sites and constraining catalytic efficiency [17, 18, 19]. Moreover, certain MOFs suffer from insufficient stability in aqueous environments, which are commonly required for biological assays [20, 21].

The stability and oxidative performance of MOFs can be further enhanced by encapsulating highly redox‐active species, such as polyoxometalates(POMs), within their confined pore environments [22, 23, 24]. Representative POMs, including phosphomolybdic acid H_3_(PMo_12_O_40_) [25], tungstophosphoric acid [26], and silicotungstic acid [27]‐ possess electronically versatile frameworks, which redox chemistry and considerable structural flexibility. These attributes enable them to serve as inorganic building blocks that interact strongly with MOFs through coordination bonds or hydrogen bonding, thereby producing highly stable composite materials, commonly referred to as PMA‐incorporated MOFs [28, 29]. The diverse coordination environments available within MOF architectures further facilitate the assembly of POM‐MOF hybrids, giving rise to unique structures driven by synergistic interactions between the POM clusters and the host framework, which in turn lead to enhanced catalytic performance. [30, 31]. Moreover, strong electronic coupling between the POM and MOF components can induce reversible redox cycling between Mo^+6^ and Mo^+5^ species, accompanied by the formation of oxygen vacancies [32, 33, 34]. Owing to these cooperative effects, POM‐MOF composite materials have been widely exploited in photosynthesis, electrocatalysis, and a range of oxidative transformation reactions [35].

3D printing technology, as a novel additive manufacturing method, offers various technical advantages, such as enhanced design freedom, rapid prototyping, customized production, and reduced material waste [36, 37]. It enables the creation of intricate and complex structures that are challenging or even impossible to achieve using traditional manufacturing approaches [38]. Metal additive manufacturing inherently generates substrates with microscale surface roughness and complex surface topologies, which can promote material adhesion and enhance interfacial stability. These characteristics make 3D printing an attractive platform for the immobilization of catalytic and enzyme‐mimetic materials, where mechanical robustness, ease of handling, and reusability are essential for practical applications [36, 38, 39].

Despite these advantages, direct fabrication of MOFs via 3D printing remains challenging, as most MOF syntheses require solvothermal or hydrothermal reaction conditions that are incompatible with direct MOF‐based ink or hydrogel printing processes [40]. Consequently, additive manufacturing is more effectively employed as an enabling platform for catalyst immobilization rather than as a direct route for MOF fabrication. Immobilizing nanozymes on structured solid supports provides an effective strategy to overcome these limitations, enabling repeated use and facilitating integration into sensing devices and flow‐based systems [18]. Metal substrates fabricated via additive manufacturing offer additional advantages, including chemical resistance, thermal stability, and compatibility with solvothermal growth conditions required for MOF synthesis, making them particularly suitable for developing robust and reusable MOF‐based nanozyme platforms.

Given these aforementioned advantages, we aimed to employ 3D printing techniques for the fabrication of MOF‐Heteropolyacid for enzyme‐mimicking activity. However, it is essential to note that most metal–organic framework (MOF) syntheses typically require specialized hydrothermal reaction conditions. Consequently, these conditions may not be compatible with direct MOF‐based hydrogel synthesis using 3D printing technology, thereby limiting the manufacturing [40].

To the best of our knowledge, this is the first report of synthesizing an in situ Fe‐based MOF (Fe‐BTC)‐Heteropoly acid composite on 3D printed metal substrates as nanozymes and for Enhanced Peroxidase‐Mimic Activity and Ultra‐Sensitive Glucose Detection. The composites (stated as Fe‐BTC‐PMA) have shown significantly higher activity compared to the parent MOF (Fe‐BTC) and PMA. The material was well characterized using various techniques, and the reaction mechanism for nanozyme activity was established. This exceptional catalytic activity can originate from the catalyst's unique structure of Heteropolyacid confined MOF, which can be explained by X‐ray absorption spectroscopy (XAS) (XANES and EXAFS) and X‐ray photoelectron spectroscopy (XPS). In addition, a detailed kinetic study was performed to gain insight into the reaction mechanism and the interaction between the nanozyme and various reactants. This state‐of‐the‐art technology can be potentially translated on an industrial scale for the real‐time detection of glucose and various biomedical applications due to the very high reusability of metal substrates.

## 2 Results and Discussion

**SCHEME 1:**
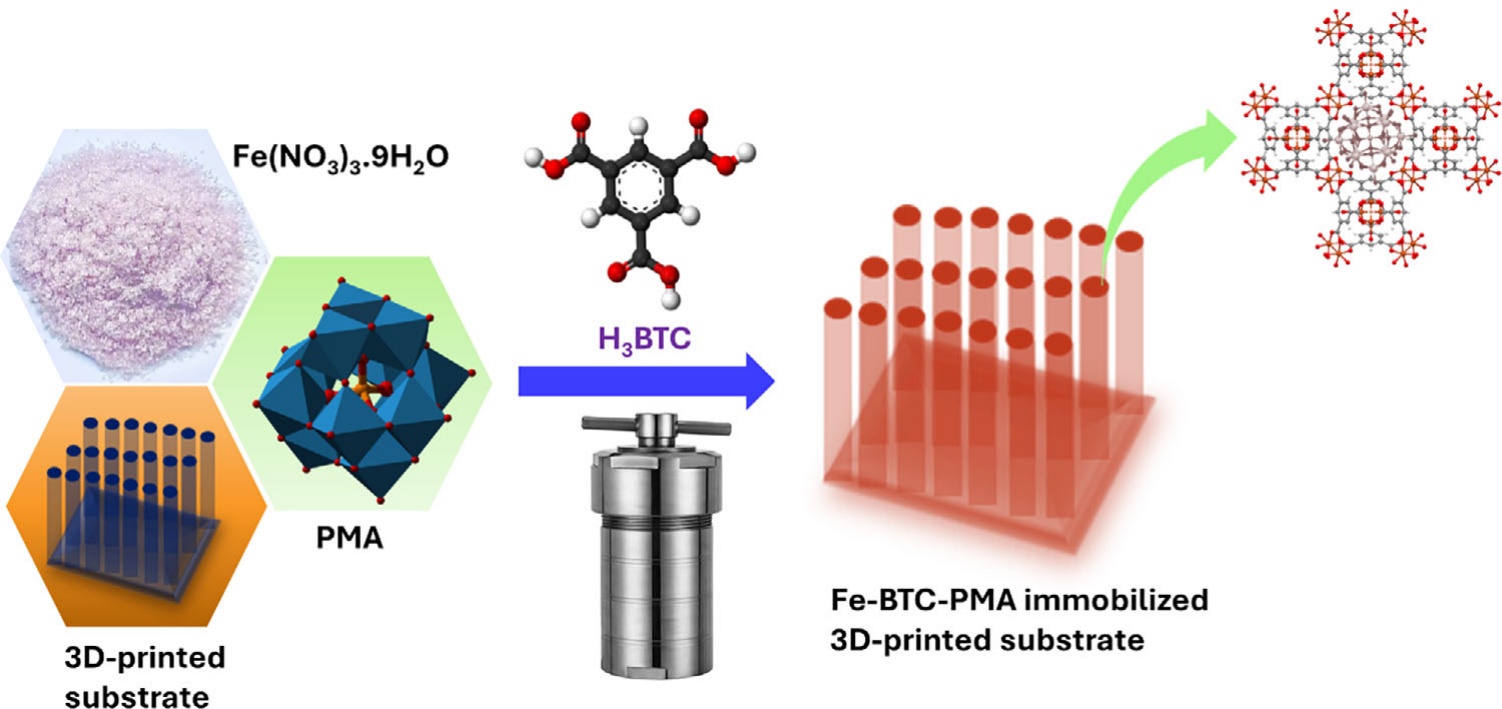
Fe‐BTC‐PMA synthesis via encapsulation of PMA in Fe‐BTC MOF matrix.

Fe‐BTC and Fe‐BTC‐PMA were synthesized via a modified solvothermal method based on the procedure described by Koley et al. [41] (Scheme 1) (see Supporting Information, Experimental Section for details). Keggin‐type PMA was incorporated into Fe‐BTC through an in situ synthesis approach, ensuring uniform dispersion of PMA while preserving its structural and compositional integrity [42]. The deposition of Fe‐BTC‐PMA was carried out by placing the 3D‐printed Ti─Al─V metal substrates into the autoclave during the Fe‐BTC‐PMA synthesis process. Afterward, the substrates were washed three times with boiling ethanol for 3 h each, followed by drying in an oven at 60°C for 24 h.

### 2.1 Morphology Analysis

**FIGURE 1:**
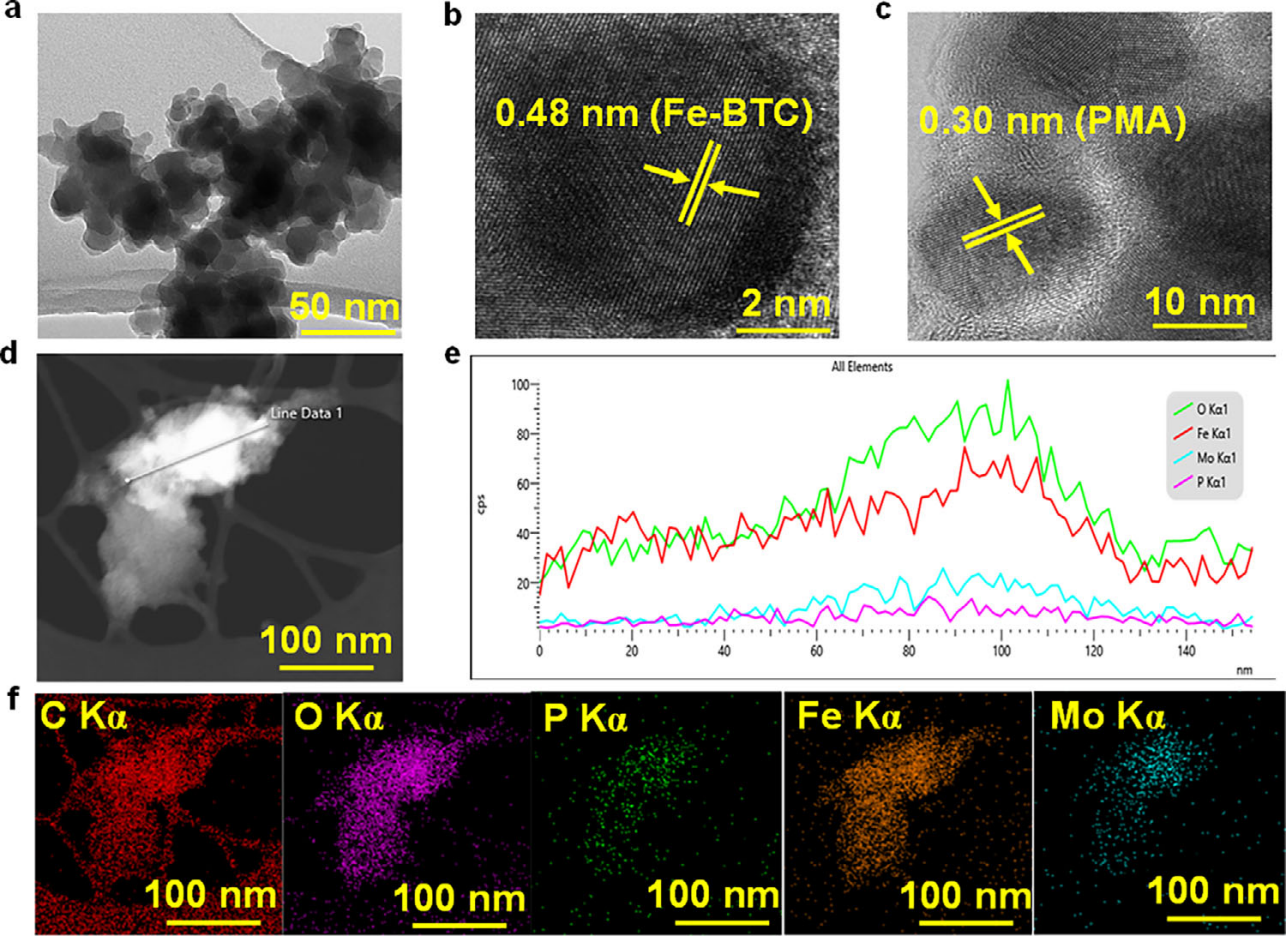
(a–c) HR‐TEM of Fe‐BTC‐PMA composite. (d,e) This line mapping analysis interpreted that PMA is incorporated in the Fe‐BTC MOF pore. (f) HAADF STEM elemental images of Fe‐BTC‐PMA.

TEM analyses of Fe‐BTC, PMA, and Fe‐BTC‐PMA (Figure 1a–c) reveal their morphological characteristics. The HR‐TEM image (Figure 1b,c) confirms the coexistence of Fe‐BTC and PMA within the Fe‐BTC‐PMA composite catalyst. Furthermore, HR‐TEM analysis suggests that the MOF phase envelops the PMA crystal lattice, indicating successful incorporation of PMA into the MOF framework. This finding aligns with previous reports demonstrating the encapsulation of polyoxometalates, such as phosphomolybdic acid (PMA), within Cu‐BTC MOF using a similar synthetic strategy [41].

TEM images illustrate a cloud‐like morphology, confirming that the structural characteristics of Fe‐BTC are preserved in the Fe‐BTC‐PMA composite. The pristine Fe‐BTC exhibits a similar morphology, consistent with earlier studies. STEM images (Figure 1f) further validate the uniform dispersion of Mo, P, Fe, O, and C within the composite, indicating homogeneous distribution of PMA in the MOF matrix.

Additionally, the presence of lattice fringes corresponding to PMA (0.30 nm) and Fe‐BTC in Fe‐BTC‐PMA suggests that both components retain their structural integrity within the composite. EDX line mapping (Figure 1d,e) provides further evidence of PMA encapsulation within the MOF pores, showing Fe and O primarily at the periphery, while Mo and P are concentrated in the interior. This elemental distribution strongly supports the successful integration of PMA into the MOF structure. Similar observations have been reported for the encapsulation of polyoxometalates within MOF pores, as demonstrated through EDX line scans for Cu‐BTC‐PMA in previous studies.

**FIGURE 2:**
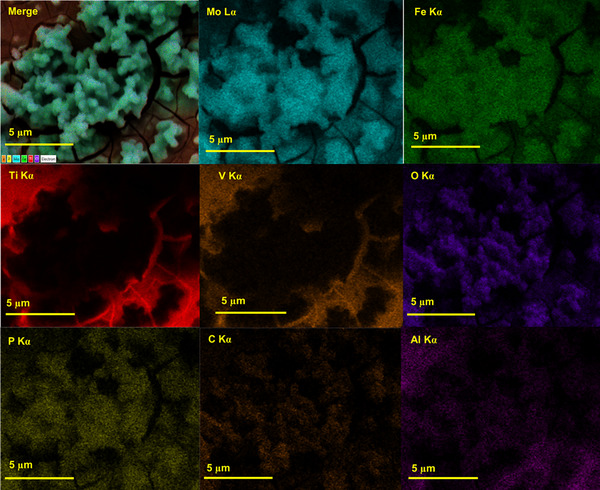
SEM‐elemental mapping of Fe‐BTC‐PMA deposited 3D printed substrates (Ti‐V‐Al).

The morphology of the Fe‐BTC‐PMA nanozyme and its deposition on 3D‐printed metal substrates were examined using SEM imaging. SEM images (Figure S3b,c) of the bare 3D‐printed metallic substrate (Ti─Al─V) and the Fe‐BTC‐PMA‐coated substrate confirm the uniform deposition of Fe‐BTC‐PMA. Additionally, EDX mapping (Figure 2) reveals the homogeneous distribution of Fe within the deposited material, while the presence of Ti, Al, and V from the underlying 3D‐printed substrate is also evident.

### 2.2 Structure Analysis

**FIGURE 3:**
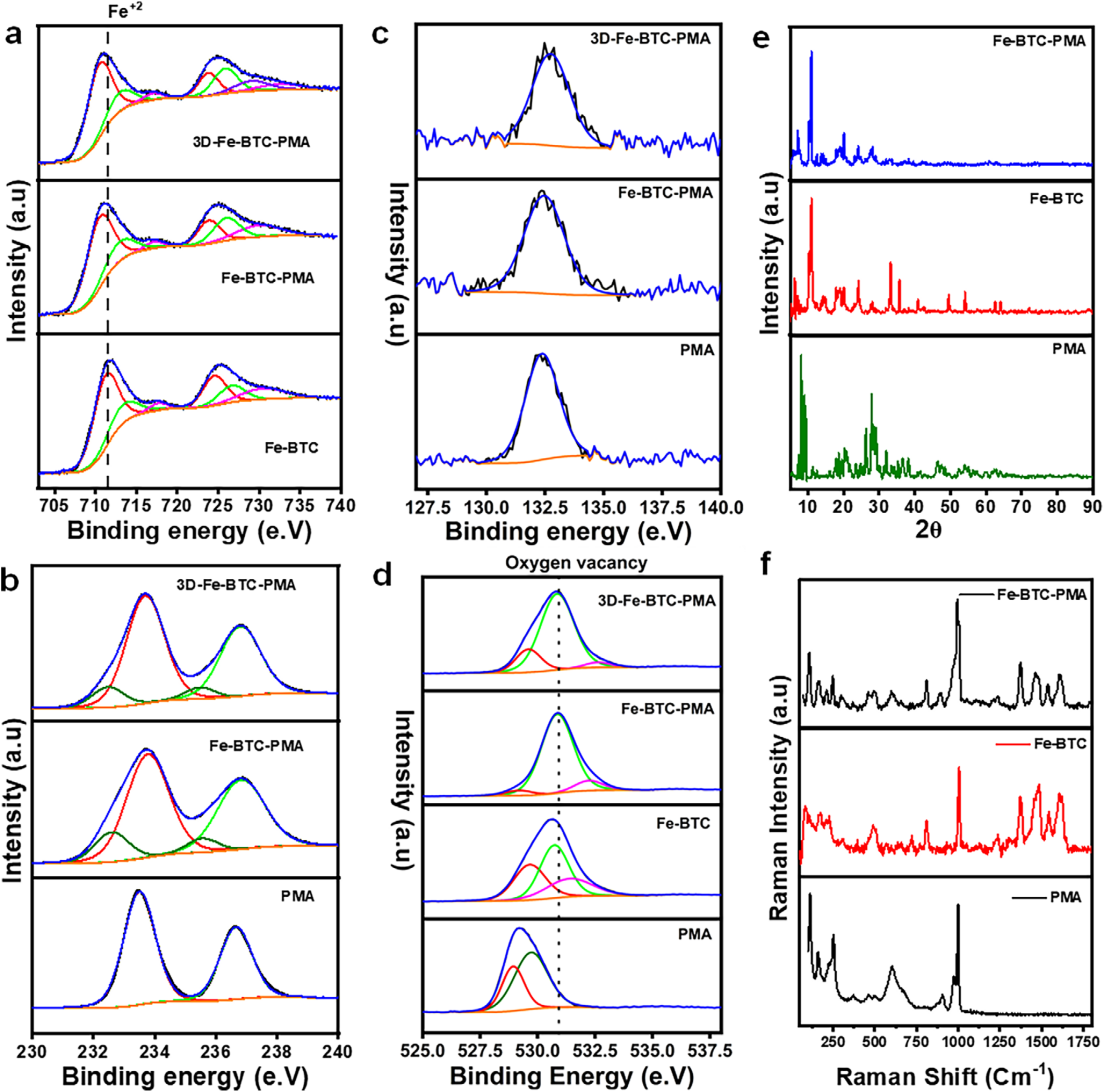
XPS scan of (a) Fe 2p, (b) Mo 3d, (c) P 2p, and (d) O 1s in PMA, Fe‐BTC, Fe‐BTC‐PMA, 3D‐Fe‐BTC‐PMA; (e) PXRD and (f) Raman spectra of Fe‐BTC show the characteristic peak of MOF, which also exists in the Fe‐BTC‐PMA composite, suggesting that the MOF structure remains intact in the Fe‐BTC‐PMA composite.

Powder X‐ray diffraction (PXRD) patterns (Figure 3e) of the synthesized materials, including Fe‐BTC and Fe‐BTC‐PMA, closely resemble the simulated Fe‐BTC pattern, confirming the retention of this crystalline phase [43]. Notably, the composite material (Fe‐BTC‐PMA) exhibits peak positions and intensities similar to those of the parent MOF (Fe‐BTC) and PMA, suggesting the uniform incorporation of PMA within the Fe‐BTC framework.

Similarly, FT‐IR spectra (Figure S3a) of the Fe‐BTC‐PMA catalyst display characteristic vibrational bands at 1065, 960, and 870 cm^−1^ [44], corresponding to the stretching modes of P‐O_a_ (O_a_ = oxygen coordinated to three Mo atoms and P), Mo‐O_t_ (O_t_ = terminal oxygen), and Mo‐O_b_‐Mo (O_b_ = corner‐sharing bridging oxygen), respectively [45]. Additionally, a broad band at 790 cm^−1^ is attributed to the Mo‐O_c_‐Mo stretching vibration. These spectral features indicate that the heteropoly Keggin structure remains intact following its incorporation within the MOF, preserving both its primary and secondary structural integrity.

The surface areas of all catalysts were measured and are presented in Table S1 and Figure S4a. Among the as‐synthesized materials, Fe‐BTC exhibits the highest surface area (1,074 m^2^ g^−^^1^). The decrease in surface area observed for Fe‐BTC following PMA incorporation is mainly due to the partial occupation and blockage of the MOF pores by the bulky PMA molecules, which limit access to adsorption sites. Additionally, strong host–guest interactions between PMA and the MOF framework may further reduce pore connectivity and overall accessible porosity. The reduction in pore volume is primarily a result of internal cavity filling by PMA species, which decreases the free void space within the framework, while pore blocking and possible framework distortions induced by host–guest interactions further contribute to the loss of accessible volume. The BJH pore size distribution, along with the average pore diameter and pore volume for the different materials, is shown in Figure S4b and Table S1. The observed increase in average pore diameter after PMA incorporation can be explained by the preferential filling or blocking of smaller pores, shifting the pore size distribution toward larger channels, along with minor framework relaxation caused by host–guest interactions.

To investigate the chemical state and surface composition, X‐ray photoelectron spectroscopy (XPS) analysis was conducted. The high‐resolution Fe 2p XPS spectrum of Fe‐BTC (Figure 3a) exhibits prominent peaks at 710.2 and 724.2 eV, corresponding to Fe 2p_3/2_ and Fe 2p_1/2_, respectively, along with satellite peaks at 718.2 and 730.6 eV [46]. Upon forming the Fe‐BTC‐PMA composite, these peaks shift to higher binding energies compared to Fe‐BTC, indicating electronic interactions between Fe‐BTC and PMA [47]. The Fe 2p_1/2_ peak in Fe‐BTC consists of Fe^2+^ at 723.6 eV, Fe^3+^ at 725.4 eV, and a satellite feature at 731.6 eV. In Fe‐BTC‐PMA, the deconvoluted Fe^2+^ and Fe^3+^ peaks within the Fe 2p_1/2_ region also shift toward higher binding energies, consistent with the behavior observed for the Fe 2p_3/2_ peak.

As depicted in Figure 3d, the O 1s spectrum of pure PMA can be deconvoluted into two peaks at 529.7 and 528.9 eV, corresponding to hydroxyl oxygen and lattice oxygen, respectively. The O 1s spectrum of Fe‐BTC alone displays three distinct peaks at 529.6, 530.9, and 533.1 eV, attributed to lattice oxygen, hydroxyl oxygen, and oxygen vacancies, respectively [7]. In the Fe‐BTC‐PMA composite, the presence of these three‐oxygen species is maintained, but their binding energies shift to higher values compared to those in pure PMA and Fe‐BTC. Notably, the Fe‐BTC‐PMA composite exhibits a greater surface concentration of oxygen vacancies than Fe‐BTC alone.

Furthermore, compared to pure PMA, the Fe‐BTC‐PMA composite displays a decrease in the binding energy of P 2p (Figure 3c), with Fe‐BTC‐PMA exhibiting the most significant shift. These binding energy variations suggest a strong electronic interaction between Fe‐BTC and PMA, further supporting the successful integration of PMA into the MOF structure.

Following deposition onto 3D‐printed metal substrates, the Fe 2p, P 2p, and O 1s peak positions remain similar to those observed in bare Fe‐BTC‐PMA (Figure 3a–d).

A cyclic voltammetry (CV) study was conducted to examine the redox behavior of three catalysts, PMA, Fe‐BTC, and Fe‐BTC‐PMA as shown in Figure S5. The redox cycle of Fe‐BTC displayed the characteristic Fe(III) + e− ↔ Fe(II) transition [48]. In contrast, the redox profile of Fe‐BTC‐PMA differs significantly from that of Fe‐BTC, resembling a combination of FeSO_4_ and Fe‐BTC, which contain both Fe^2^^+^ and Fe^3^^+^ species [49]. In Fe‐BTC‐PMA, the iron oxidation state can fluctuate between Fe^2^^+^ and Fe. The integration of the PMA cluster into the Fe‐BTC framework modifies the redox environment of iron in the Fe‐BTC‐PMA material, potentially enhancing its nanozyme activity. Similarly, Koley et al. previously reported that incorporating PMA into the Cu‐BTC catalyst altered its redox behavior, with the presence of Cu^2^^+^ species being advantageous for oxidation reactions [41]. Given that Fe‐BTC‐PMA exhibits a similar redox pattern to FeSO_4_, it can be inferred that the presence of Fe^2^^+^ species likely promotes Fenton‐like oxidation and facilitates the Fe^3^^+^/Fe^2^^+^ redox cycle.

### 2.3 EXAFS Analysis

**FIGURE 4:**
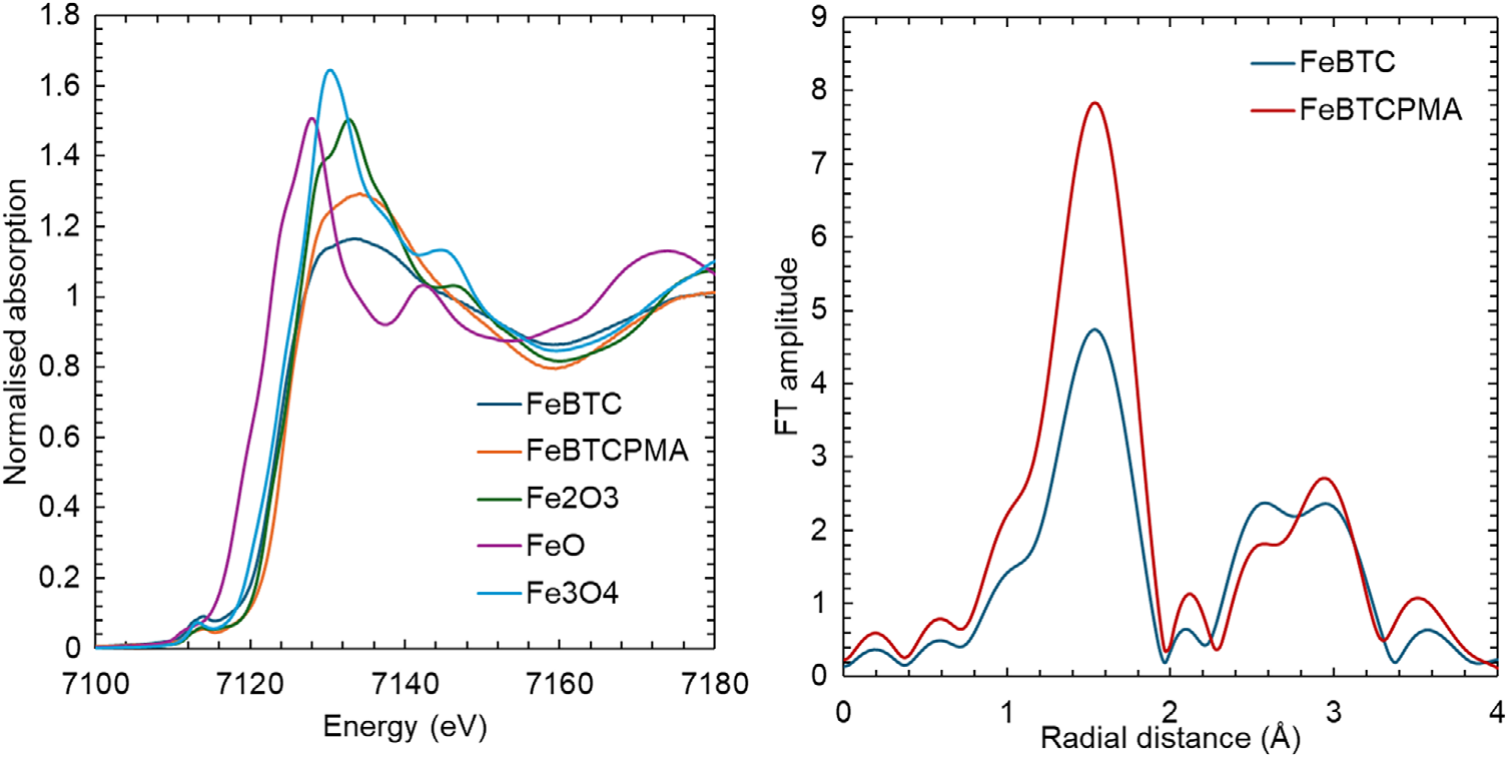
XANES spectra (a) and FT magnitudes (b) for Fe K‐edge EXAFS oscillations for FeBTC and FeBTCPMA.

To obtain comprehensive insights into the electronic configuration, oxidation state, and local coordination structure of Fe species, X‐ray absorption spectroscopy (XAS) measurements were systematically performed at the Fe K‐edge, encompassing both X‐ray Absorption Near Edge Structure (XANES) and Extended X‐ray Absorption Fine Structure (EXAFS) analyses (Figure 4; Figure S6). The k^3^‐weighted EXAFS data were analyzed meticulously using a curve‐fitting approach in both R‐space and k‐space to extract quantitative structural parameters such as bond distances, coordination numbers, and disorder factors. Figure 4a presents the Fe K‐edge XANES spectra of pristine Fe‐BTC and PMA‐modified Fe‐BTC (Fe‐BTC‐PMA), along with representative Fe oxide standards‐Fe_2_O_3_ (Fe^3+^), Fe_3_O_4_ (Fe^2+^/Fe^3+^ mixed), and FeO (Fe^2+^)‐for reference. The main absorption edge for both Fe‐BTC and Fe‐BTC‐PMA is centered around 7124 eV, corresponding to the characteristic 1s → 4p dipole‐allowed transition of Fe^3+^ species [50]. The close overlap of these edge positions with that of Fe_2_O_3_, together with the absence of a distinct shift toward lower energy, unambiguously confirms that the majority of Fe centers in both catalysts exist in a trivalent oxidation state [51]. Moreover, the pre‐edge feature located near 7113 eV, which originates from the 1s → 3d quadrupole transition, exhibits comparable intensity and shape for both samples. This indicates that the local coordination geometry around Fe remains largely octahedral and centrosymmetric upon PMA incorporation, ruling out any substantial distortion or reduction of Fe sites during the composite formation. Beyond oxidation state analysis, subtle changes in XANES spectral intensity provide evidence for chemical interaction between PMA and the Fe nodes. The slightly changed white‐line intensity for Fe‐BTC‐PMA compared to Fe‐BTC suggests an increase in local electron density perturbation and hybridization between Fe 3d and O 2p orbitals, likely arising from interfacial bonding with Mo‐O clusters. This interfacial coupling is further elucidated in the EXAFS region (Figure 4b), where the Fourier‐transformed magnitude spectra exhibit distinct structural differences between the two samples.

Both Fe‐BTC and Fe‐BTC‐PMA display a dominant first‐shell peak at approximately 1.5 Å (uncorrected for phase shift), characteristic of Fe─O coordination. However, the amplitude of this first‐shell feature is noticeably higher in Fe‐BTC‐PMA, implying a stronger backscattering contribution and a higher degree of local structural order around Fe. The absence of significant peaks beyond ∼3 Å indicates that Fe atoms are highly dispersed without any contribution from Fe–Fe scattering paths, consistent with the preservation of isolated Fe sites rather than formation of iron oxide aggregates. Quantitative curve fitting of the EXAFS data (Figure S6 and Table S4) provides further confirmation. The coordination number (CN) for Fe─O increases from that in pristine Fe‐BTC (CN = 6) to approximately 8.2 in Fe‐BTC‐PMA, confirming enhanced oxygen coordination in the PMA‐modified framework. This structural evolution can be ascribed to the formation of Fe─O─Mo interfacial bonds, in which oxygen atoms bridge Fe^3^^+^ centers from the Fe‐BTC framework and Mo^6^^+^ centers from the PMA anion. Such Fe─O─Mo linkages establish an efficient interfacial electron‐transfer channel, promoting electronic delocalization between Fe 3d and Mo 4d orbitals. This charge redistribution leads to partial electron depletion around Fe and corresponding enrichment on Mo, resulting in a more favorable redox potential alignment. Consequently, the Fe sites become more catalytically active and capable of accelerating redox reactions involving reactive oxygen species. This atomic‐level electronic modulation accounts for the markedly enhanced peroxidase‐like activity and ultra‐sensitive glucose oxidation performance of Fe‐BTC‐PMA, as compared to pristine Fe‐BTC [41].

Overall, the combined XANES and EXAFS analyses clearly demonstrate that PMA incorporation does not alter the oxidation state of Fe but substantially modifies its local coordination environment and electronic structure through Fe─O─Mo interfacial coupling. These structural and electronic synergies provide a rational explanation for the superior catalytic and sensing behaviour of the Fe‐BTC‐PMA composite

### 2.4 Raman Spectroscopy

Raman spectroscopy was employed to investigate the structural features of the MOF and its composite with polyoxometalate. As shown in Figure 3f, the peaks at 746 and 831 cm^−^^1^ correspond to the stretching and bending vibrations of C─H bonds. The bands observed at 1546 and 1461 cm^−^^1^ are attributed to the symmetrical and asymmetrical vibrations of the C─O_2_ functional groups, respectively [52]. A distinct peak at 1000 cm^−^^1^, characteristic of PMA, is retained in the Fe‐BTC‐PMA composite [41]. Additionally, signals at 968 and 179 cm^−^^1^ indicate the presence of Mo─O─Mo bonding [53]. The retention of all characteristic peaks associated with the Fe‐BTC MOF in the composite confirms that the MOF framework remains intact following its integration with PMA.

### 2.5 Catalytic Performance and Glucose Detection

#### 2.5.1 Peroxidase‐Like Activity

**FIGURE 5:**
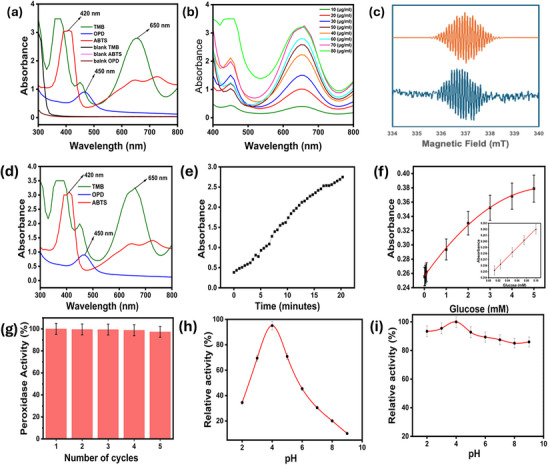
(a) Absorbance spectra of ABTS (0.6 mm), OPD (0.6 mm), and TMB (0.8 mm) recorded in pH 4.0 buffer (100 mm) containing Fe‐BTC‐PMA (100 µg mL^−^^1^) at 25°C after 10 min of incubation. (b) Absorbance spectra of the TMB substrate with increasing concentrations of Fe‐BTC‐PMA (10–80 µg mL^−^^1^) after 10 min of incubation. (c) EPR spectrum (blue) showing the presence of TMB^+^. (ν = 9.442 GHz, power = 2 mW, modulation = 0.01 mT, modulation frequency = 100 Hz). Fit of spectrum (red) using parameters given in text using EasySpin [54]. (d) UV–vis absorbance spectra of ABTS (0.6 mM), OPD (0.6 mm), and TMB (0.6 mm) in pH 4 buffer (100 mm) containing 100 µg mL^−^^1^ Fe‐BTC‐PMA deposited 3D printed metal substrate at 25°C after 10 min of incubation. (e) Time‐dependent absorbance changes at 653 nm for TMB. (f) Glucose concentration response curve for glucose detection using glucose oxidase and Fe‐BTC‐PMA 3D MS (inset: linear calibration plot for glucose). (g) Reusability of the Fe‐BTC‐PMA 3D MS for nanozyme activity. (h) Effect of pH (1 to 10) on the peroxidase‐like activity of Fe‐BTC‐PMA at constant concentrations of TMB, and H_2_O_2_, and (i) corresponding pH stability of Fe‐BTC‐PMA showing relative catalytic activity retained across a wide pH range.

Following the successful characterization of the morphologies and chemical/electronic structures of Fe‐BTC‐PMA, its enzyme‐mimetic sensing performance, including activity, selectivity, and stability, was further investigated. The peroxidase‐like activity of PMA, Fe‐BTC, and Fe‐BTC‐PMA catalysts was assessed based on their ability to catalyze the oxidation of 3,3',5,5'‐tetramethylbenzidine (TMB) substrate in the presence of H_2_O_2_ within 10 min, as shown in Figure S8. Among the tested catalysts, Fe‐BTC‐PMA exhibited the highest activity, evidenced by the development of a blue color with maximum absorbance at 650 nm. In contrast, neither PMA nor H_2_O_2_ alone produced a significant color change, confirming that the observed reaction was specifically catalyzed by Fe‐BTC‐PMA. The relative catalytic activities of the materials followed the order: Fe‐BTC‐PMA > Fe‐BTC > PMA, as depicted in Figure S8. Time‐dependent kinetic studies further demonstrated that the Fe‐BTC‐PMA catalyst induced a more rapid increase in TMB absorbance compared to the other catalysts (Figure 5a), highlighting its superior peroxidase‐like activity. Given its strong performance, the ability of Fe‐BTC‐PMA to catalyze the oxidation of additional substrates, such as ABTS and OPD, was also explored. The reaction solutions of TMB, ABTS, and OPD, treated with Fe‐BTC‐PMA and H_2_O_2_ for 10 min, displayed characteristic color changes and corresponding UV–vis spectra, confirming successful oxidation of all three substrates (Figure 5a). Further experiments were conducted to evaluate the effect of Fe‐BTC‐PMA concentration on TMB oxidation. As shown in Figure 5b, the reaction rate increased with catalyst concentration, reaching a plateau at 80 µg mL^−^^1^, beyond which no significant enhancement was observed. Kinetic analysis over a 0–20‐min interval for varying concentrations of Fe‐BTC‐PMA also revealed that higher concentrations (up to 80 µg mL^−^^1^) led to increased absorbance over time, confirming the concentration‐dependent catalytic efficiency of Fe‐BTC‐PMA (Figure 5e).

#### 2.5.2 Peroxidase‐Mimetic Activity and Reusability of Fe‐BTC‐PMA‐Modified 3D‐Printed Metal Substrates

Following the immobilization of the Fe‐BTC‐PMA nanozyme onto 3D‐printed metal substrates (denoted as Fe‐BTC‐PMA 3D MS), its peroxidase‐like catalytic performance was evaluated using TMB, ABTS, and OPD as chromogenic substrates. The results demonstrate that Fe‐BTC‐PMA retains high catalytic efficiency after deposition onto the 3D‐printed platforms (Figure 5d). Time‐dependent kinetic studies of TMB oxidation catalyzed by Fe‐BTC‐PMA 3D MS were performed over a 0–20 min interval, revealing a gradual increase in absorbance with reaction time (Figure 5e). The reusability of the immobilized Fe‐BTC‐PMA nanozyme was systematically assessed over five consecutive catalytic cycles using the same 3D‐printed metal substrate (Figure 5g). After each cycle, the substrate was thoroughly rinsed three times with Milli‐Q water to remove residual species and then dried at 40°C for 24 h prior to reuse. The nanozyme exhibited excellent operational stability, retaining ∼91% of its initial catalytic activity after five cycles, with only a marginal decline from the first use. This high level of activity retention highlights the strong immobilization of Fe‐BTC‐PMA on the substrate and the intrinsic structural robustness of the nanozyme, underscoring its suitability for repeated and practical peroxidase‐mimicking applications.

To further assess the stability of the reused nanozyme, XPS and FT‐IR analyses of the 3D‐printed substrate were performed after 5 days of cyclic reactions (Figure S7). The XPS results indicated that the oxidation states of Fe remained unchanged, while the FT‐IR spectra of the reused nanozyme were consistent with those of the fresh nanozyme. These findings confirm that the structural integrity of the Fe‐BTC‐PMA nanozyme was preserved after repeated use. The use of 3D‐printed metal substrates provides a promising platform for designing and producing efficient, reusable catalytic systems. This approach not only enhances the functionality of nanozyme‐based applications but also offers potential for waste reduction through improved substrate reusability.

### 2.6 In Situ EPR Spectroscopy

Electron spin resonance (ESR) spectroscopy was employed to investigate the peroxidase‐like catalytic activity of Fe‐BTC‐PMA by probing hydroxyl radical (OH**^·^**) generation from H_2_O_2_ at pH 7.4 using 5,5‐dimethyl‐1‐pyrroline‐N‐oxide (DMPO) as a spin‐trapping agent. The characteristic 1:2:2:1 quartet signal typically associated with the DMPO‐OH adduct was not observed as shown in Figure 5c, but rather a complex 18‐line spectrum centred at g = 2.004, typical of the TMB radical cation [55]. Fitting of this spectrum revealed hyperfine splitting parameters almost identical to those proposed by Josephy et al. $a_{NH_{2}}^{N}=8.96\;MHz,$ $a_{NH_{2}}^{H}=9.04\;MHz$, $a_{ring}^{H}=2.96\;MHz$. The presence of TMB**^·^** ^+^ and absence of a DMPO based radical does not rule out OH**^·^** generation but indicates that in the complex reaction mixture, it might be hard to trap ROS when there are so many radical species present.

### 2.7 Evaluation of Catalytic Activity Under Varying Temperature and pH Conditions

**FIGURE 6:**
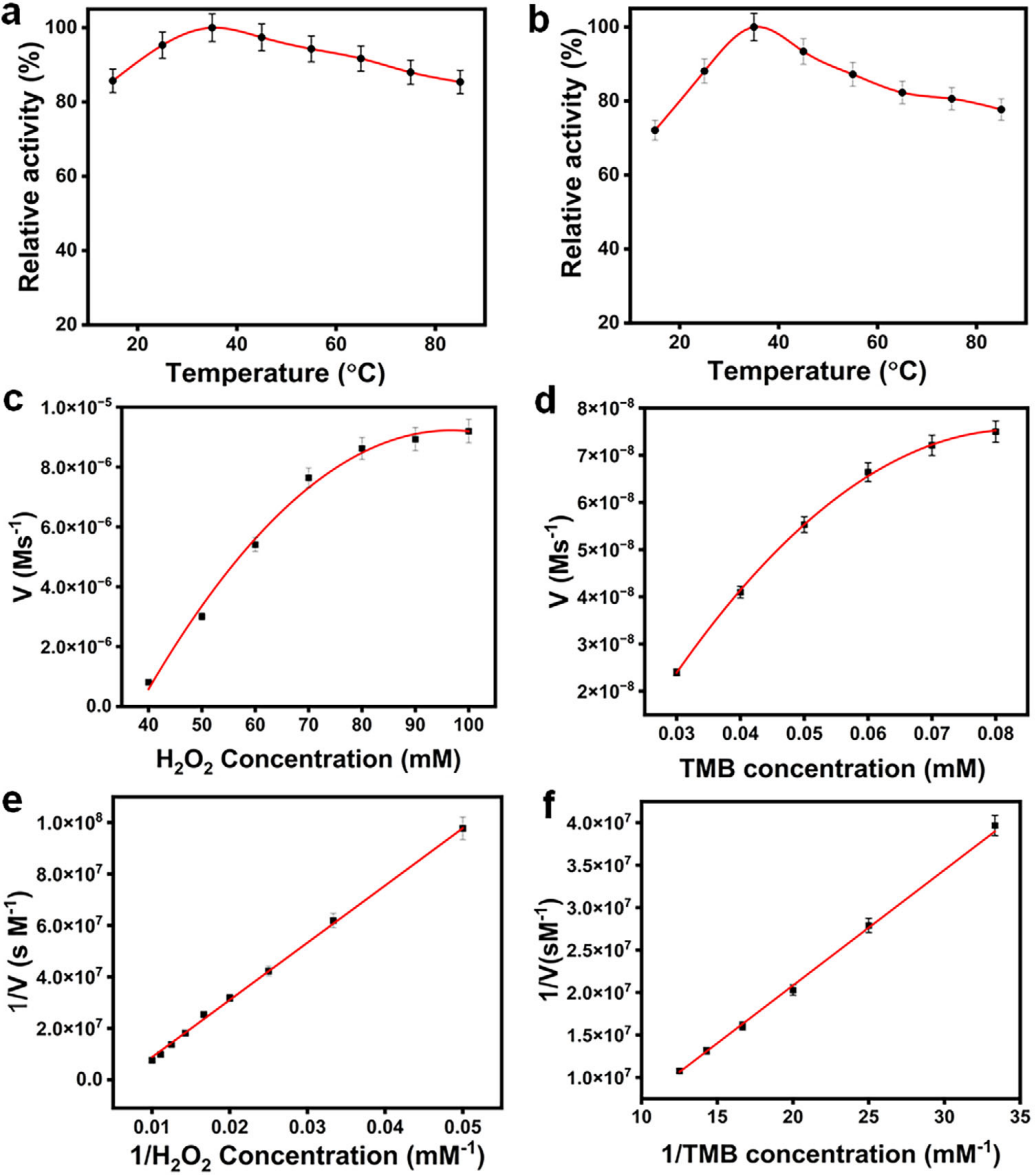
(a) Temperature‐dependent peroxidase‐like activity of Fe‐BTC‐PMA measured at pH 4 over a temperature range of 15–85°C, and (b) corresponding thermal stability evaluated by relative catalytic activity over the same temperature range. (c) Steady‐state enzyme‐like kinetics of the Fe‐BTC‐PMA catalyst based on normalized Michaelis‐Menten plots (d) Activity data at a fixed TMB concentration of 800 µL and varying H_2_O_2_ concentrations (20‐100 mm), and (e) activity data at a fixed H_2_O_2_ concentration of 100 mM and varying TMB concentrations (30‐80 µm). The corresponding Lineweaver‐Burk plots for H_2_O_2_ and (f) TMB are also presented.

The influence of pH on the peroxidase‐like activity of Fe‐BTC‐PMA was investigated over a pH range of 2 to 9, while the concentrations of Fe‐BTC‐PMA, TMB, and H_2_O_2_ were kept constant. As shown in Figure 5h,i, the activity optimization curve exhibited a sharp peak at pH 4.0, with a significant decrease in absorbance observed under both more alkaline and strongly acidic conditions. This behavior is typical of certain peroxidase mimetics and closely parallels that of natural peroxidases. The relative activity–pH profile indicates that Fe‐BTC‐PMA exhibits optimal catalytic performance for TMB oxidation under mildly acidic conditions (pH 4.0). In addition, the temperature dependence of the peroxidase‐like activity of Fe‐BTC‐PMA was examined at a fixed pH of 4.0 by varying the reaction temperature from 15 to 85°C, as shown in Figure 6a,b. All experiments were performed in triplicate, with error bars included to indicate reproducibility. The catalytic activity gradually increased from 85% to 99% as the temperature rose from 15°C to 40°C. Beyond this, the activity declined slightly, reaching 80% at 85°C. Typically, natural peroxidases exhibit significant loss of activity at elevated temperatures, especially above 70°C. Notably, Fe‐BTC‐PMA retained approximately 80% of its catalytic activity at 85°C, demonstrating enhanced stability and catalytic efficiency compared to natural peroxidases.

The stability of Fe‐BTC‐PMA across different pH levels was also assessed, as illustrated in Figure 5i. The catalyst retained 90% of its relative activity over a broad pH range from 2.0 to 9.0. In contrast, natural enzymes such as horseradish peroxidase (HRP) typically lose activity at pH levels below 5 or at temperatures above 70°C. However, Fe‐BTC‐PMA displayed remarkable stability, maintaining catalytic activity across a wide temperature range (15°C to 85°C) and pH spectrum. These results suggest that Fe‐BTC‐PMA exhibits superior stability and catalytic performance compared to natural peroxidases, making it a promising candidate for applications under diverse environmental conditions.

### 2.8 Insights into the Kinetics and Mechanism of Peroxidase‐Mimetic Catalysis

Kinetic studies were conducted to assess the Michaelis‐Menten behavior of the Fe‐BTC‐PMA catalyst toward TMB and H_2_O_2_. As illustrated in Figure 6c,d, well‐defined Michaelis–Menten curves were obtained over the investigated concentration ranges of both substrates. The kinetic parameters, including the Michaelis constant (K_m_) and maximum reaction velocity (V_m_), were extracted from the fitted plots and are summarized in Table S5. For benchmarking purposes, the catalytic performance of Fe‐BTC‐PMA was compared with that of horseradish peroxidase (HRP). Notably, Fe‐BTC‐PMA exhibited a substantially lower K_m_ value for TMB (0.126 mm) than HRP (0.434 mm) [56], indicating a higher affinity of the catalyst toward TMB oxidation. This result suggests that Fe‐BTC‐PMA exhibits superior catalytic activity toward TMB compared to HRP. Additionally, Lineweaver–Burk plots were generated for both H_2_O_2_ and TMB substrates, as illustrated in Figure 6e,f.

The Fe‐BTC‐PMA composite exhibits significantly enhanced peroxidase‐like activity compared to pristine Fe‐BTC, enabling efficient oxidation of TMB in the presence of H_2_O_2_. This enhancement can be attributed to the synergistic interaction between Fe centers in Fe‐BTC and the polyoxomolybdate (PMA) clusters. In this system, Fe sites in the Fe‐BTC framework can undergo redox cycling between Fe^3^^+^ and Fe^2^^+^ states, analogous to the classical Fenton reaction, to catalyze the decomposition of H_2_O_2_ and generate reactive hydroxyl radicals (•OH) [57]. Concurrently, Mo centers in the PMA clusters facilitate electron transfer, stabilizing the redox‐active Fe species and enhancing the formation of •OH radicals [58]. The generated •OH radicals act as potent oxidizing agents, rapidly converting colorless TMB into its oxidized, blue‐colored product (oxTMB).

Although the catalytic behavior of Fe‐BTC‐PMA shares similarities with classical Fenton‐type systems, the experimental evidence suggests that the peroxidase‐like activity does not rely solely on freely diffusing hydroxyl radicals. The absence of a characteristic DMPO–OH signal in the EPR spectra, together with the detection of TMB radical cations, indicates that substrate oxidation likely proceeds predominantly via surface‐mediated redox pathways involving adsorbed reactive intermediates and high‐valent metal–oxo species.

**SCHEME 2:**
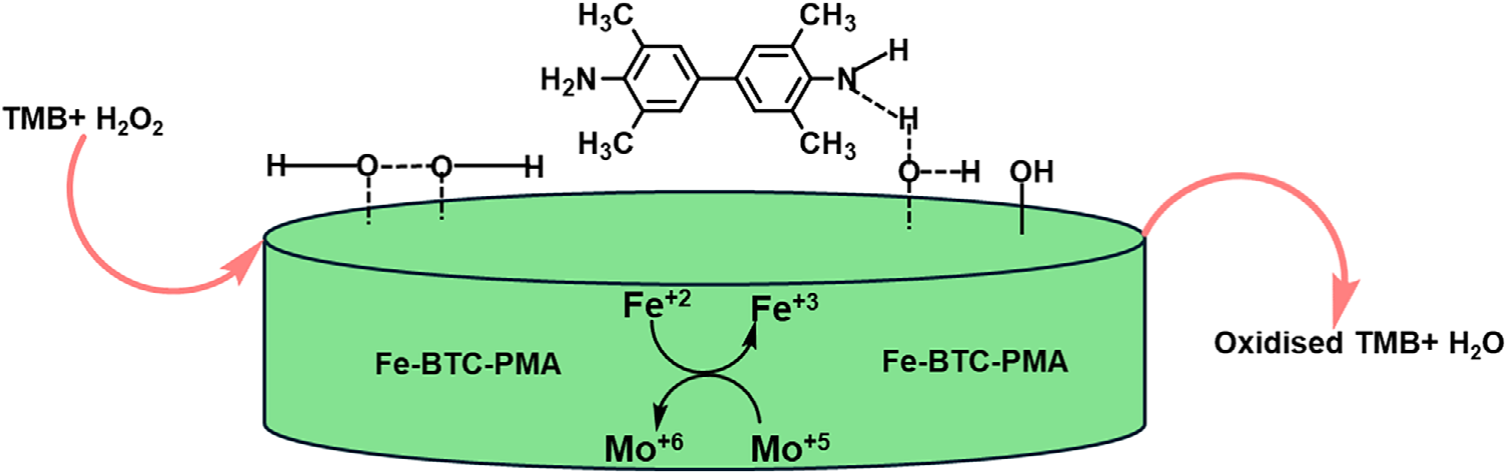
Proposed peroxidase‐mimetic catalytic mechanism at iron and molybdenum. centers for TMB oxidation.

The strong electronic coupling between Fe centers in the MOF framework and Mo centers in the PMA clusters facilitates efficient electron transfer, stabilizing redox‐active sites and promoting rapid substrate oxidation. Oxygen vacancies further contribute by enhancing H_2_O_2_ activation at the catalyst surface. Consequently, the superior peroxidase‐like performance of Fe‐BTC‐PMA can be attributed to a synergistic surface‐confined catalytic mechanism rather than a purely homogeneous Fenton reaction, consistent with the observed pH dependence, EPR results, and kinetic behavior. The overall catalytic mechanism can be summarized as follows:

Schematic illustration (Scheme 2) of the peroxidase‐like catalytic mechanism of Fe‐BTC‐PMA. Although Fe K‐edge XANES confirms that Fe^3^^+^ is the dominant oxidation state under ex situ conditions, the reaction proceeds via a dynamic, surface‐confined Fe^3^^+^/Fe^2^^+^ redox cycle generated in situ during H_2_O_2_ activation. Strong electronic coupling between Fe‐BTC and PMA facilitates the transient reduction of Fe^3^^+^ to catalytically active Fe^2^^+^ species, enabling Fenton‐like oxidation through surface‐bound reactive intermediates rather than bulk homogeneous hydroxyl radical pathways.

1. **Activation of H_2_O_2_**:

Fe^2+^−BTC+H_2_O_2_ = Fe^3+^−BTC+^.^OH+OH^−^

**2. Regeneration of Fe^2^^+^ by PMA**:

Fe^3+^−BTC + Mo (V)−PMA = Fe^2+^−BTC +Mo (VI)−PMA

**3. TMB Oxidation**:

TMB+^.^OH = oxTMB

The enhanced peroxidase‐like activity of the Fe‐BTC‐PMA nanozyme can be ascribed to the synergistic contribution of active Fe sites in Fe‐BTC and the presence of phosphomolybdic acid (PMA). Both components participate in Fenton‐like reactions with H_2_O_2_, thereby amplifying catalytic activity. Moreover, oxygen vacancies within the Fe‐BTC‐PMA framework are proposed to play a crucial role in activating its peroxidase function. XPS analysis (Table S2) confirmed a high density of surface oxygen vacancies, which are believed to facilitate the generation of reactive oxygen species that subsequently oxidize TMB into oxygenated products. The catalyst thus benefits from a dual mechanism: Fe^3^^+^ centers in Fe‐BTC and PMA act as active species, while oxygen vacancies promote reactive oxygen species formation. Furthermore, XPS, XANES, and EXAFS analyses revealed an electronic interaction between Fe in the MOF and Mo in PMA, which likely enhances the overall catalytic performance of the composite. By contrast, the individual components‐Fe‐BTC and commercial PMA‐exhibited considerably lower peroxidase‐like activity. These results demonstrate that the Fe─Mo interactions combined with oxygen vacancies endow Fe‐BTC‐PMA with superior nanozyme activity compared to its separate counterparts. This synergy highlights the potential of Fe‐BTC‐PMA as a robust nanozyme platform for colorimetric biosensing and related catalytic applications.

### 2.9 Computational Studies

In continuation of our recent computational investigations on the nanozymatic behavior of degraded MIL‐88B [7], the present study explores the peroxidase‐like catalytic activity of Fe and Mo active centers embedded within Fe‐BTC and Fe‐BTC‐PMA porous metal‐organic frameworks (MOFs). In our previous work, we developed computational models of iron oxide active sites derived from degraded MIL‐88B to elucidate the mechanistic features governing the enzyme‐like peroxidation of TMB [7]. In a subsequent study, we provided theoretical insight into the catalytic functions of Cu and Mo sites in Cu‐BTC and Cu‐BTC‐PMA frameworks for the oxidation of 5‐hydroxymethylfurfural (HMF) to 2,5‐diformylfuran (DFF) [41].

Building upon these findings, the current work focuses on constructing molecular models to describe the peroxidation of TMB catalyzed by Fe‐BTC and Fe‐BTC‐PMA. Previous quantum‐chemical studies have proposed that the peroxidase‐mimetic mechanism is initiated by the dissociative adsorption of hydrogen peroxide (H_2_O_2_) on iron oxide surfaces, followed by successive reductions of hydroxylated intermediates by TMB. The dissociation of H_2_O_2_ has been reported to proceed feasibly at Fe centers of iron oxide clusters, characterized by high adsorption energies that render the removal of surface‐bound species by TMB energetically demanding [59, 60]. To extend this mechanistic understanding, we employed density functional theory (DFT) [61] and quantum theory of atoms in molecules (QTAIM) [62]. analyses to quantify the nature and strength of the interactions between H_2_O_2_ and the metal active sites in Fe‐BTC and Fe‐BTC‐PMA. The calculated adsorption free energies revealed that H_2_O_2_ binds more strongly to Fe‐BTC‐PMA (ΔE_ads_ = ‐75.11 kcal mol^−^^1^) than to Fe‐BTC (ΔE_ads_ = ‐54.43 kcal mol^−^^1^), suggesting a more favorable adsorption and dissociation process on the Fe‐BTC‐PMA surface. Topological analysis of the electron density distribution was subsequently performed for both catalyst models, and the corresponding QTAIM molecular graphs (MGs) are presented in Figure S10. The computed QTAIM parameters at key bond critical points (BCPs) and ring critical points (RCPs) are summarized in Table S6. In both catalysts, the interaction of H_2_O_2_ with the metal centers generates two stable RCPs that significantly influence bond strength and charge distribution. A comparative assessment of QTAIM data indicates that the Fe and Mo adsorption sites exhibit comparable electron density characteristics, with ρ_*BCP*_ values in the range of 0.01‐0.05 a.u. and ρ_*RCP*_ values of 0.007‐0.02 a.u. The higher catalytic performance of Fe‐BTC‐PMA in peroxidation reaction can therefore be attributed primarily to the more favorable adsorption thermodynamics of H_2_O_2_ at its active sites, rather than to notable differences in electronic structure.

### 2.10 Optical Detection of Glucose

**FIGURE 7:**
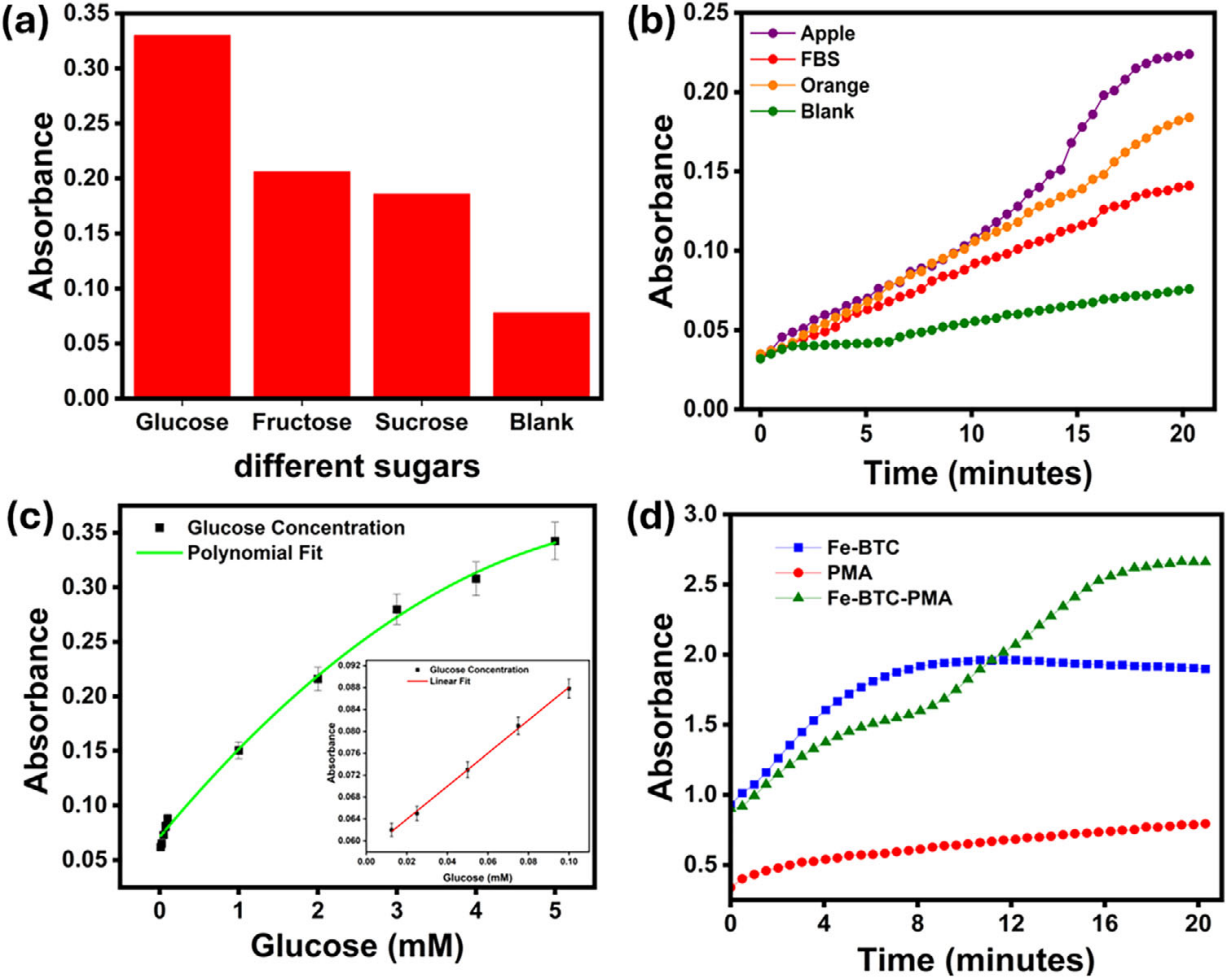
(a) Selectivity test for glucose detection. (b) Time‐dependent absorbance changes at 653 nm for different samples (spiked FBS, orange juice, and apple juice) after incubation with GOx and Fe‐BTC‐PMA. The samples‐orange juice and apple juice‐were diluted 50‐fold, 500‐fold, and 2500‐fold, respectively, while spiked FBS was prepared at a concentration of 4 mm. (c) Glucose concentration‐response curve for detection using GOx and Fe‐BTC‐PMA. (d) Time‐dependent absorbance changes at 653 nm for TMB catalyzed by PMA, Fe‐BTC, and Fe‐BTC‐PMA.

Glucose detection is widely recognized as essential in numerous biomedical applications as well as in the food industry. Leveraging the intrinsic peroxidase‐like activity of the Fe‐BTC‐PMA nanozyme, we developed a straightforward colorimetric approach for the quantitative determination of glucose in diverse samples. In this system, glucose oxidase (GOx) catalyzes the oxidation of glucose in the presence of molecular oxygen, generating gluconic acid and hydrogen peroxide (H_2_O_2_). The generated H_2_O_2_ then reacts with the TMB substrate in the presence of the Fe‐BTC‐PMA nanozyme, resulting in a blue‐colored reaction mixture. This method provides a reliable means to measure glucose content. The selectivity of the method was further assessed using other sugars, including fructose and sucrose, and the results were compared with those for glucose (Figure 7a). The Fe‐BTC‐PMA nanozyme exhibited markedly higher sensitivity and selectivity toward glucose than toward fructose and sucrose. Given that the glucose concentration in the blood of a healthy individual ranges from 3 to 8 mmol L^−^^1^, fetal bovine serum (FBS) with a glucose concentration of 4 mmol L^−^^1^ was used as a representative real sample. Based on the calibration curve (inset, Figure 7c), the glucose concentration in the spiked FBS sample was measured to be 3.67 mmol L^−^^1^, closely matching the spiked value of 4.00 mmol L^−^^1^. Additionally, this method was successfully applied to detect glucose concentrations in various fruit juices, such as apple and orange juice, demonstrating its reliability for determining glucose levels in diverse real‐world samples (Figure 7b).

Exploiting the peroxidase‐like activity of the Fe‐BTC‐PMA deposited on a 3D‐printed metal substrate, a simple and effective colorimetric strategy was developed for the quantitative determination of glucose in diverse samples. In this system, glucose oxidase (GOx) catalyzes the oxidation of glucose in the presence of molecular oxygen, generating gluconic acid and hydrogen peroxide (H_2_O_2_). The generated H_2_O_2_ subsequently reacts with the TMB substrate on the Fe‐BTC‐PMA‐deposited 3D‐printed metal substrate, resulting in the formation of a blue‐colored reaction mixture. This approach offers a reliable means to measure glucose content. The relationship between glucose concentration and absorbance is depicted in Figure 5f. As shown, absorbance increased linearly with glucose concentration over the range of 1–100 µm. Based on the 3σ/k criterion, the calculated limit of detection was 3.33 µm, indicating that concentrations below this value cannot be statistically distinguished from the blank.

### 2.11 Role of 3D Printing in Catalyst Immobilization and Reusability

The immobilization of Fe‐BTC‐PMA on additively manufactured metal substrates benefits directly from the intrinsic surface characteristics of metal additive manufacturing. Previous studies have demonstrated that surface chemistry, roughness, and microstructural features of solid supports play a decisive role in the nucleation, adhesion, and mechanical stability of MOF films grown on substrates [63, 64]. Compared to smooth planar or conventionally machined substrates, the roughened and topologically complex surfaces generated by layer‐by‐layer metal additive manufacturing provide a higher density of nucleation sites and enhanced mechanical interlocking, which collectively promote robust MOF anchoring.

Fe‐BTC‐based materials have previously been reported as surface‐bound films while retaining their crystallinity and functional integrity, supporting the feasibility of substrate‐assisted MOF growth strategies [44]. In the present system, the additively manufactured metal substrate acts as a mechanically stable scaffold for the in situ growth of the Fe‐BTC‐PMA composite, enabling strong adhesion of the nanozyme layer and minimizing catalyst detachment during operation. In addition to physical roughness, the strong adhesion and catalytic enhancement of the Fe‐BTC‐PMA nanozyme on the Ti─Al─V substrate can be attributed to interfacial chemical interactions. Titanium alloys spontaneously form a stable native oxide layer (predominantly TiO_2_) under ambient conditions. This oxide surface is enriched with hydroxylated Ti─OH groups, which can act as coordination or hydrogen‐bonding sites for MOF precursors during solvothermal growth. Specifically, the Ti─OH/Ti─O surface functionalities facilitate interfacial bonding with iron‐oxo clusters and carboxylate linkers, promoting the nucleation and growth of Fe‐BTC directly on the metallic scaffold. Such Ti─O─Fe interfacial interactions improve MOF anchoring and suppress delamination under catalytic operation. Furthermore, the intimate electronic coupling between the conductive Ti─Al─V support and the Fe‐BTC‐PMA framework can promote interfacial charge redistribution, facilitating faster electron transfer during the peroxidase‐mimicking redox process. This synergistic substrate–MOF interaction contributes to the enhanced catalytic efficiency and durability of the integrated nanozyme system. This is quantitatively reflected in the excellent operational stability of the immobilized system, which retains approximately 91% of its initial peroxidase‐like activity after five consecutive catalytic cycles, following simple washing and drying between uses. Such stability is difficult to achieve with powder nanozymes, which often suffer from catalyst loss, aggregation, and performance degradation during repeated recovery steps.

The effectiveness of the immobilized architecture is further supported by the kinetic and sensing performance of the Fe‐BTC‐PMA system. Despite partial pore occupation by PMA, as evidenced by the reduction in BET surface area relative to pristine Fe‐BTC (≈ 1,074 m^2^ g^−^^1^ for Fe‐BTC), the composite exhibits a low Michaelis‐Menten constant for TMB (Km = 0.126 mm), which is substantially lower than that of horseradish peroxidase (≈ 0.434 mm) under comparable conditions. This indicates that immobilization on the additively manufactured substrate does not compromise substrate accessibility and, instead, enables efficient utilization of active sites. In glucose sensing, the immobilized Fe‐BTC‐PMA nanozyme achieves a linear detection range of 1–100 µM with a limit of detection of 3.33 µm, demonstrating that the solid‐supported configuration maintains high analytical sensitivity while offering superior handling and reuse. Although the 3D‐printed Ti─Al─V substrate provides robust MOF growth and excellent reusability, the cost of large‐scale production may exceed that of conventional polymeric supports. Optimization of printing parameters or exploration of alternative alloys could help mitigate these costs. Moreover, while preliminary cyclic and operational tests indicate strong stability, the long‐term performance under extended use or storage has not yet been systematically evaluated. Addressing these factors in future studies will be important to ensure the practical scalability and sustained catalytic efficiency of the nanozyme platform.

## 3 Conclusion

In conclusion, we establish a mechanistically informed approach to enhancing nanozyme activity through the in situ confinement of heteropoly acid species within an Fe‐based MOF grown on 3D‐printed metal substrates. The Fe‐BTC‐PMA hybrid exhibits markedly improved peroxidase‐like activity, arising from synergistic electronic interactions between PMA and the Fe‐BTC framework. Advanced spectroscopic investigations, including XANES, EXAFS, and XPS, reveal substantial modulation of the Fe coordination environment and charge redistribution induced by PMA incorporation, which collectively promote efficient electron transfer and activation of peroxide substrates. These experimental observations are further supported by detailed kinetic analyses, in situ EPR measurements, and DFT calculations, which together elucidate the reaction pathway and identify the key active sites responsible for the enhanced catalytic performance. The combined experimental–theoretical framework provides direct evidence that nanoscale confinement and interfacial electronic coupling are central to achieving superior nanozyme activity. In addition to mechanistic insight, the hybrid system exhibits excellent structural stability and reusability, underscoring the robustness of the catalytic architecture. As a result, the Fe‐BTC‐PMA system represents a promising candidate for next‐generation glucose sensors suitable for real‐time monitoring and point‐of‐care diagnostics. More broadly, the design principles established here provide a versatile framework for engineering MOF‐based nanozymes on advanced architecture, opening new opportunities for their translation into biomedical diagnostics, environmental monitoring, and industrial sensing technologies.

## Conflicts of Interest

The authors declare no conflicts of interest.

## Supporting information




**Supporting File**: advs74743‐sup‐0001‐SuppMat.docx.

## Data Availability

The data that support the findings of this study are available from the corresponding author upon reasonable request.
