# Supplementary material for: 3D‐Printed Metal‐Supported MOF‐Heteropoly Acid Nanozyme for High‐Performance Peroxidase‐Mimic Activity and Ultra‐Sensitive Glucose Detection
Source: Adv Sci (Weinh). 2026 Mar 12;13(41):e24355. doi: 10.1002/advs.202524355 (PMC13325815; doi:10.1002/advs.202524355)
Supplement: Supplementary file 1 — Supporting File: advs74743‐sup‐0001‐SuppMat.docx. [file ADVS-13-e24355-s001.docx]

**3D-Printed Metal-Supported MOF-Heteropoly acid Nanozyme for High-Performance Peroxidase-Mimic Activity and Ultra-Sensitive Glucose Detection**

Paramita Koley*^a#^, Ranjithkumar Jakku^a#^, Subhash Chandra Shit^b^, Jang Mee Lee^a^, Guy N. L. Jameson^c^, Tayebeh Hosseinnejad^a^, Selvakannan Periasamy^a^, Deshetti Jampaiah^a^, Amrit Raj Paul^a^, Ylias Sabri^a^, and Suresh K. Bhargava*^,a^

^a^Centre for Advanced Materials & Industrial Chemistry (CAMIC), School of Applied Sciences, RMIT University, GPO Box 2476, Melbourne 3001, Australia.

^b^Department of Energy Engineering/KENTECH Institute for Environmental and Climate Technology, Korea Institute of Energy Technology (KENTECH), Naju 58330, Republic of Korea.

^c^School of Chemistry and Bio21 Molecular Science and Biotechnology Institute, The University of Melbourne, 30 Flemington Road Parkville Vic 3010, Australia.

Corresponding Authors: Paramita Koley; Email: [paramita.koley@rmit.edu.au](mailto:paramita.koley@rmit.edu.au)

Suresh K Bhargava; Email: suresh.bhargava@rmit.edu.au

#These two authors contributed equally

**Experimental section:**

**Chemicals and Solvents:** Iron(III) nitrate nonahydrate (Fe(NO₃)₃·9H₂O, ≥98%), 1,3,5-benzenetricarboxylic acid (H₃-BTC, ≥95%), and phosphomolybdic acid hydrate (H₃PMo₁₂O₄₀, PMA, ≥99%) were purchased from Sigma-Aldrich. Ethanol (≥99.5%) and distilled water were used as solvents throughout the synthesis. All chemicals and solvents were of analytical grade and used without further purification.

**Fe-BTC-PMA and Fe-BTC synthesis:** The materials were synthesized based on a reported method with slight modifications(*1*). First, Fe(NO₃)₃·9H₂O (3.0 mmol, 1.21 g) and phosphomolybdic acid (PMA, 0.55 mmol, 1.0 g) were dissolved in 12 mL of distilled water inside a Teflon liner. Next, H₃-BTC (3.0 mmol, 0.63 g) was added to the solution, and the mixture was stirred at room temperature for 30 min. The Teflon liner was then sealed in a stainless-steel autoclave and heated at 120 °C for 6 h. After the reaction, the autoclave was allowed to cool naturally to room temperature. The resulting solid product was separated by centrifugation and washed with 150 mL of ethanol to remove any unreacted H₃-BTC. The solid was further treated several times with boiling ethanol for 3 h, followed by centrifugation and drying in an oven at 60 °C for 24 h. The obtained material was denoted as Fe-BTC-PMA. For comparison, a Fe-BTC sample was also synthesized under the same conditions without adding PMA, and this product was referred to as Fe-BTC.

**Synthesis of Fe-BTC-PMA deposited 3D printed substrates:** 3D-printed metallic substrates composed of a Ti–Al–V alloy was employed. The substrates were first calcined in air at 500 °C for 3 h. During the synthesis of Fe-BTC-PMA, the calcined substrates were placed inside the autoclave. Following synthesis, the substrates were washed three times with boiling ethanol for 3 h each and then dried in an oven at 60 °C for 24 h, yielding the Fe-BTC-PMA-coated 3D-printed substrates.

**Characterization instruments:** Powder X-ray diffraction (P-XRD) patterns were obtained using a Bruker D8 ADVANCE diffractometer with Cu Kα radiation (λ = 1.5406 Å). Data were collected over a 2θ range of 10–90° in continuous scanning mode with a 0.01° step size and a scan rate of 5° min⁻¹. Transmission electron microscopy (TEM) images were acquired on a JEOL 1010 operating at 100 kV, while high-resolution TEM (HR-TEM) images and selected area electron diffraction (SAED) patterns were recorded on a JEOL 2010 at 200 kV. Catalyst morphology was examined using a FEI Verios 460L field-emission scanning electron microscope (FESEM) equipped with an ultrahigh-resolution Schottky emitter. Energy-dispersive X-ray spectroscopy (EDS) was carried out on the same instrument using an Oxford X-MaxN 20 detector at 25 kV. X-ray photoelectron spectroscopy (XPS) measurements were performed on a Thermo Scientific K-Alpha spectrometer with monochromatic Al Kα radiation (E photon = 1486.6 eV), and all binding energies were referenced to the C 1s peak at 284.8 eV. Confocal Raman spectra were collected at room temperature in the 200–1200 cm⁻¹ range using a Horiba Jobin–Yvon Lab RAM HR spectrometer equipped with a 17 mW He–Ne laser (λ = 632.8 nm). Approximately 5–10 mg of catalyst powder was spread on a glass slide for analysis. Nitrogen adsorption–desorption measurements were conducted at 77 K using a BEL Sorb II instrument (Japan) to determine BET surface area, pore volume, and pore size distribution. Prior to analysis, samples were degassed at 110 °C for 5 h, and measurements were performed overnight. FT-IR spectra were collected on a DIGILAB (USA) IR spectrometer. Electrochemical characterization of Fe-BTC and Fe-BTC-PMA was performed using cyclic voltammetry (CV) in a three-electrode system. A working electrode was prepared by mixing 10 μL of MIL-88B and DEG-500 with 90 μL of acetone solution, followed by drop-casting 30 μL of the mixture onto a polished glassy carbon electrode. After drying, the electrode was used in a setup with an Ag/AgCl reference electrode and a platinum wire counter electrode. Measurements were carried out in 0.2 M sodium acetate buffer (pH 3.6) over a potential range of –1.5 to +1.5 V, with a 10 mV step size, 10 μA current range, and a scan rate of 50 mV s⁻¹.

**Standard assay for the peroxidase catalytic activity study:**

The enzymatic activities of Fe-BTC, PMA, and Fe-BTC-PMA were evaluated at 25 °C. In a typical assay, 20.4 µL of H₂O₂ (100 mM) was added to a reaction mixture containing catalyst (100 µg/mL), TMB (97 µL, 800 µM) as the substrate, and sodium acetate buffer (200 µL, 100 mM, pH ≈ 4). The catalytic activities were also tested with OPD (600 µM) and ABTS (600 µM) as alternative substrates to assess selectivity. In this colorimetric reaction, H₂O₂ oxidizes the substrates in the presence of the catalyst, as represented by Equation 1. The characteristic absorbance of oxidized TMB at 653 nm was monitored continuously for 10 min using Fe-BTC, PMA, and Fe-BTC-PMA. To further study the concentration dependence, assays were conducted with Fe-BTC-PMA at increasing catalyst loadings (20, 40, 60, 80, 100, 120, and 140 µg/mL), and the absorbance of TMB was recorded.

Steady-state kinetic measurements were performed using 100 µg of Fe-BTC-PMA. In one set of experiments, the concentration of H₂O₂ (100 mM) was fixed while varying the concentration of TMB (200, 400, 600, 800, and 1000 µM). In a complementary study, the TMB concentration was held constant (800 µM) while varying the H₂O₂ concentration (20, 40, 60, 80, 100, and 150 mM).

Substrate (TMB, ABTS, ODP) + catalyst + H_2_O_2_ oxidized substrate + H_2_O (1)

The kinetic parameters were determined from Lineweaver–Burk plots, generated using the double-reciprocal form of the Michaelis–Menten equation, as outlined below.

$\frac{1}{V} = \frac{K_{m}}{V_{m}}\left( \frac{1}{\left[ S \right]}+\frac{1}{K_{m}} \right)$ (2)

where V is the initial velocity, *V*_m_ represents the maximal reaction velocity, [S] corresponds to the substrate concentration, and *K*_m_ is the Michaelis–Menten constant.

The pH-dependent activity of the Fe-BTC-PMA catalyst was evaluated using a series of 100 mM buffer solutions over the pH range 1.0–10.0. Glycine–HCl buffer was used for pH 1.0–2.0, acetate buffer for pH 3.0–5.0, phosphate buffer for pH 6.0–8.0, and Tris–HCl buffer for pH 9.0–10.0. Temperature-dependent studies were performed from 15 to 85 °C under the same conditions. Additionally, the stability of Fe-BTC-PMA at different pH values and temperatures was assessed by incubating the catalyst in the respective buffers for 24 h, after which its peroxidase-like activity was measured under optimized conditions (pH 4.0, 25 °C).

**Colorimetric detection of glucose**

Glucose detection was performed as follows. Glucose solutions with concentrations ranging from 1 µM to 5 mM were mixed with 100 µL of glucose oxidase (GOx, 2 mg mL⁻¹) in 100 mM phosphate-buffered saline (PBS, pH 7.4), resulting in a total reaction volume of 200 µL. The mixture was incubated at 37 °C for 30 min. After incubation, 600 µL of acetate buffer (100 mM, pH 4), 150 µL of TMB (1.2 mM), and 50 µL of Fe-BTC-PMA (2 mg mL⁻¹ in acetate buffer) were added. The reaction was further incubated at 37 °C for 10 min, and the absorbance at 653 nm was recorded.

For control experiments, 100 µL of 1 mM glucose, 5 mM fructose, and 5 mM sucrose were tested for comparison. To determine glucose in real samples such as spiked fetal bovine serum (4 mM glucose), apple juice, and grape juice, the samples were centrifuged at 12,000 rpm for 40 min. The resulting supernatants were diluted with 100 mM PBS (pH 7.4) by factors of 50, 2500, and 5000, respectively, prior to the assay.

**Determination of the Limit of Detection**

The limit of detection (LOD) was calculated according to the standard 3σ/k criterion, where σ represents the standard deviation of the blank measurements and k is the slope of the linear calibration curve. Blank samples containing all reagents except glucose were measured in triplicate (or n ≥ 5), and the corresponding standard deviation was obtained from these signals. The slope was derived from the linear regression of absorbance versus glucose concentration within the working range.

**Computational details:**

We have first determined the optimized structure of Fe-BTC and Fe-BTC-PMA MOF catalyst models at PBE0/6-31G* level of theory(*2*). It is noteworthy that PBE is particularly suited for studying interactions between molecules and metal surfaces, while also providing reliable results for bulk calculations. PBE0 functional incorporates a 3:1 ratio of Perdew–Burke–Ernzerhof (PBE) exchange energy and Hartree-Fock exchange energy, alongside the full PBE correlation energy.

In order to confirm the stationary points as minima, we have verified all real frequencies. All DFT computations have been performed using ORCA suite of programs(*3, 4*).The topological properties of electron densities were examined at the optimized structure of all designed catalyst models via using the calculated PBE0/6-31G* wave function files as inputs to AIM2000 program package(*5*).


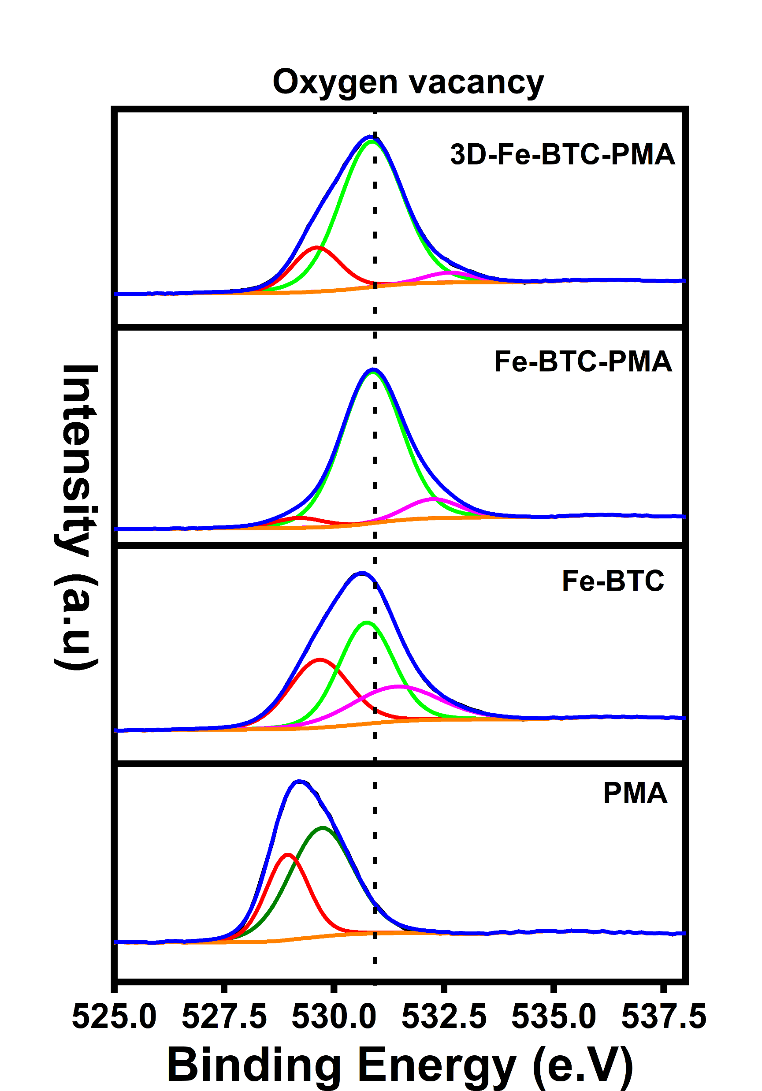


**Figure S1**: High-resolution XPS Scan of O 1s in PMA, Fe-BTC, Fe-BTC-PMA, 3D-Fe-BTC-PMA.


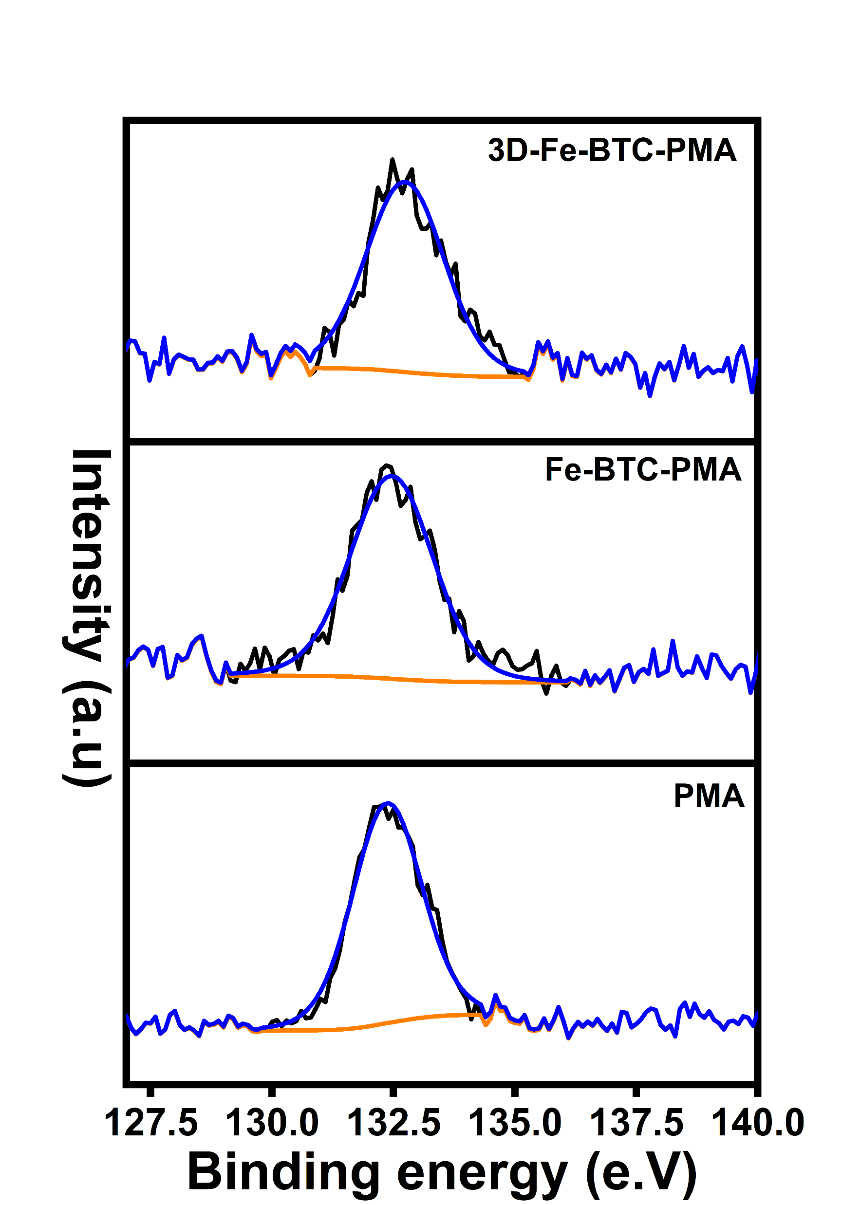


**Figure S2:** High-resolution XPS Scan of P 2p in PMA, Fe-BTC-PMA, 3D-Fe-BTC-PMA.


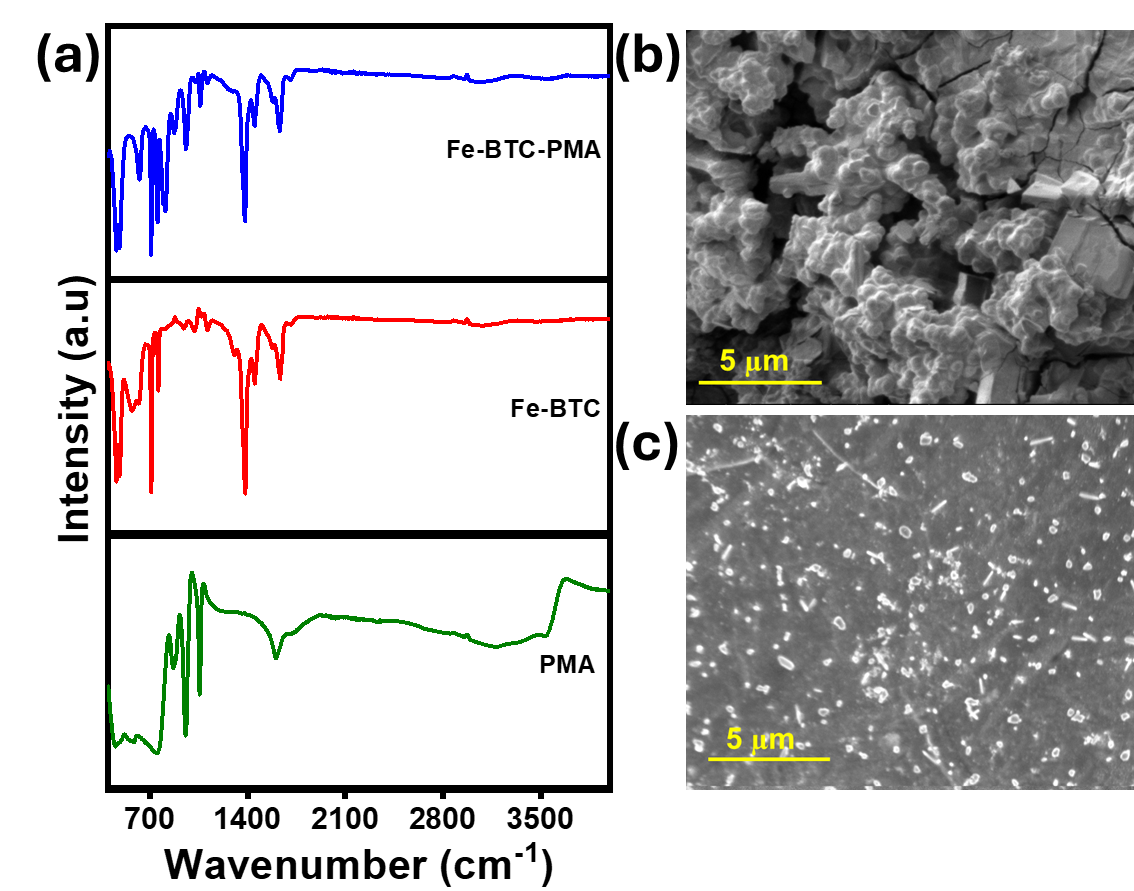


**Figure S3**. (a) FT-IR of Fe-BTC-PMA consists of both peaks of Fe-BTC and PMA. (b) Fe-BTC-PMA deposited 3D printed substrates and (c) blank calcined 3D printed substrates.


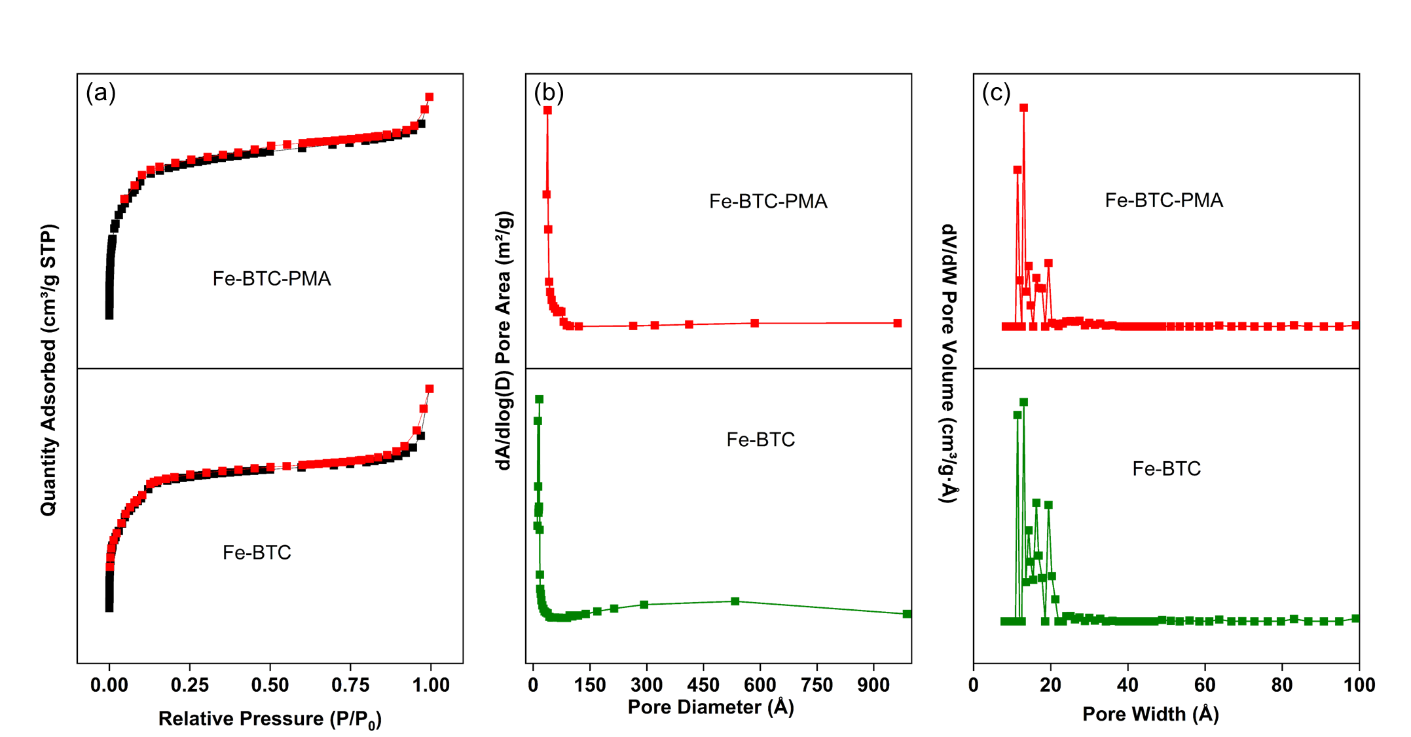


**Figure S4**: (a) N_2_ physisorption (b) BJH pore size distribution and (c) NLDFT pore size distribution for Fe-BTC, Fe-BTC-PMA.


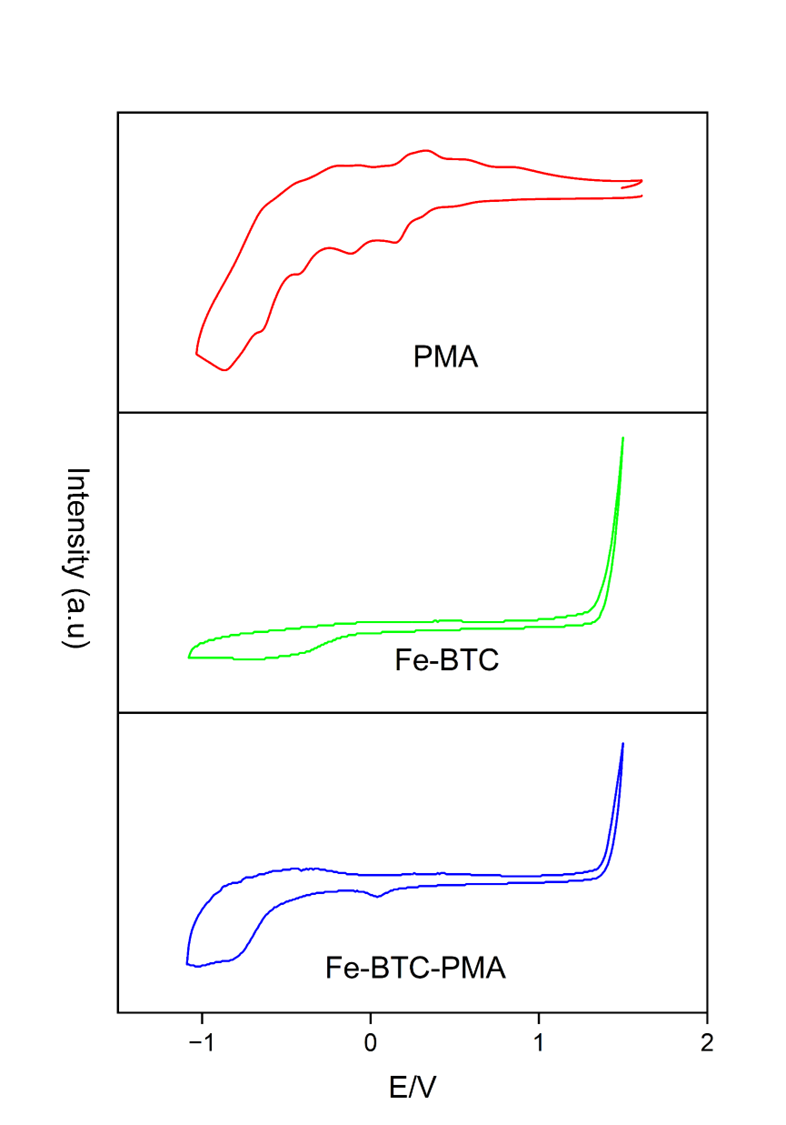


**Figure S5:** Cyclic voltammetry (CV) for PMA, Fe-BTC, Fe-BTC-PMA.


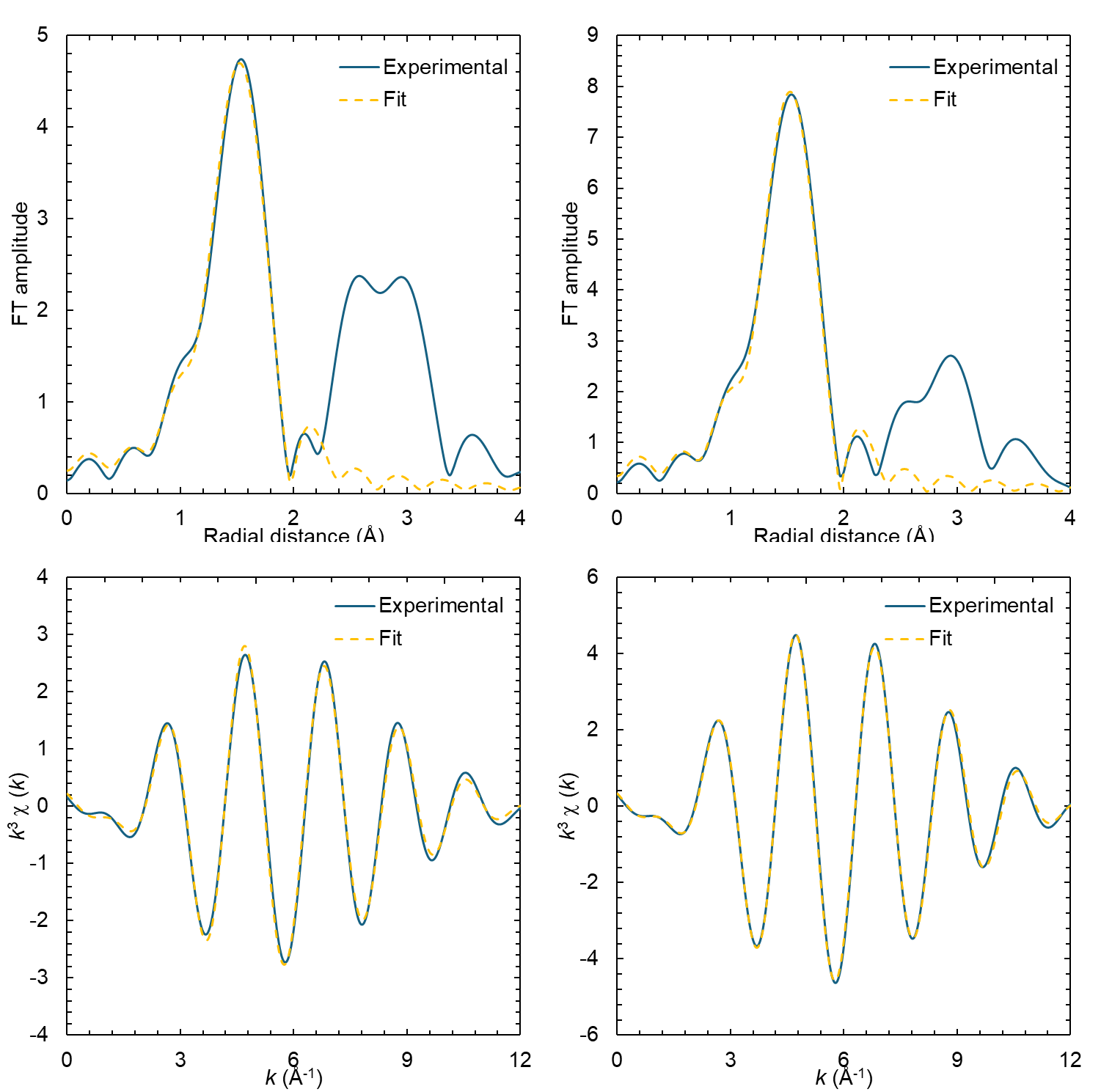


**Figure S6**. FT amplitudes and *k^3^*-weighted oscillations for Fe K-edge EXAFS spectra for Fe-BTC and Fe-BTC-PMA. The solid and dashed lines represent the experimental data and the calculated data, respectively.


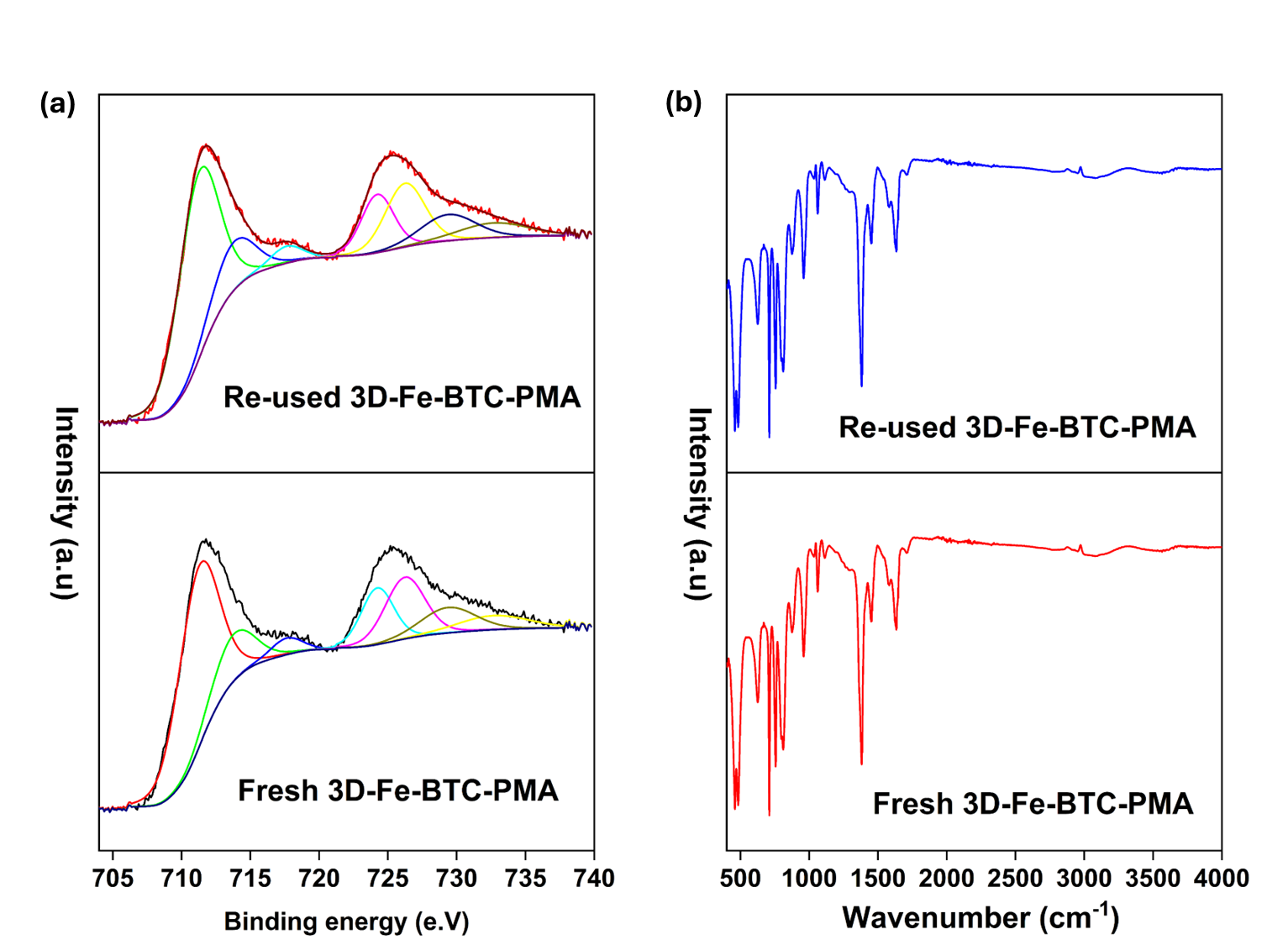


**Figure S7**: (a) XPS and (b) FT-IR of 3D-Fe-BTC-PMA reused and fresh


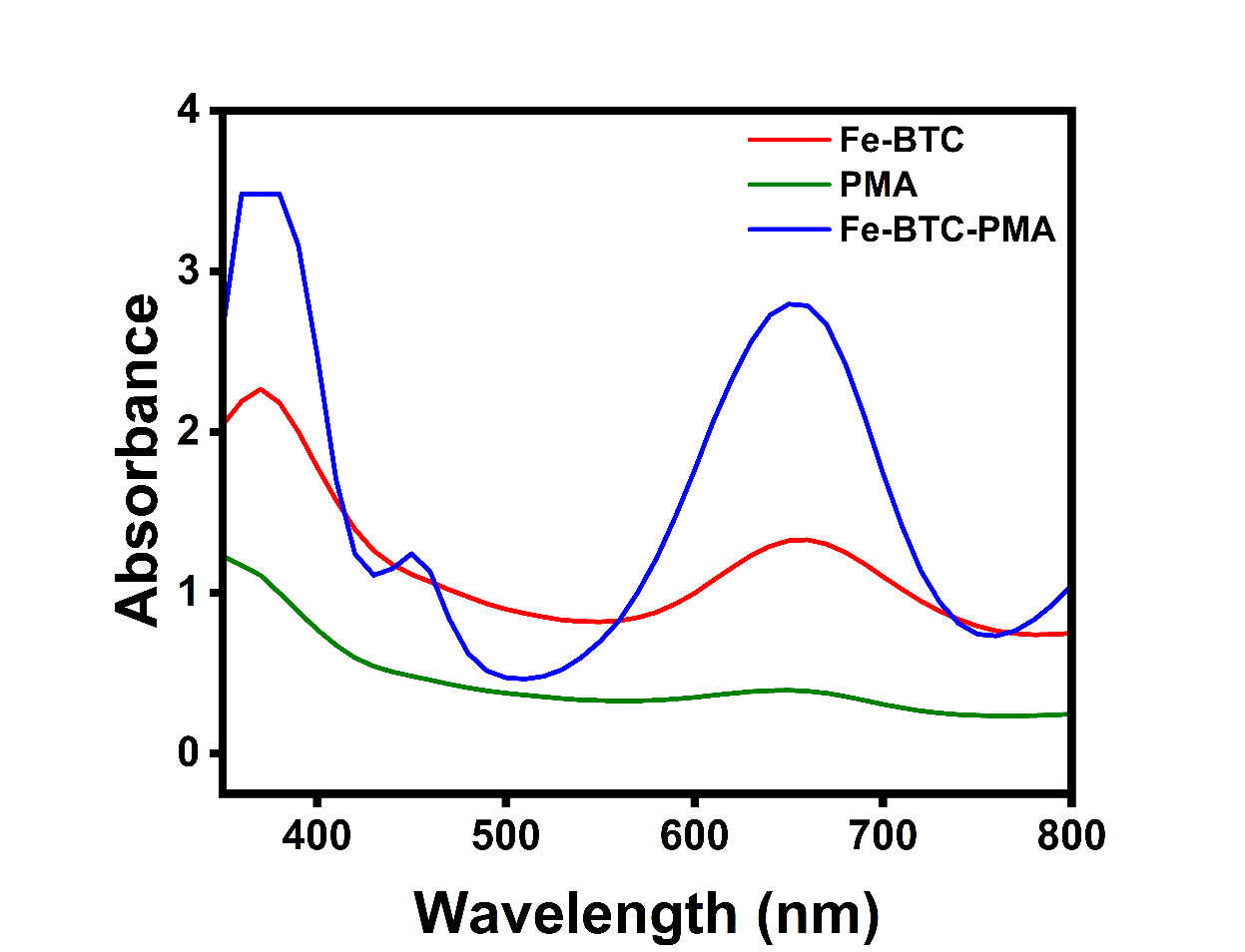


**Figure S8**: UV-vis Absorbance spectra of the peroxidase-like activity of PMA, Fe-BTC, and Fe-BTC-PMA in presence of TMB and H_2_O_2_.

**Figure S9**: Square-root (power) versus intensity for the EPR spectra of TMB**^·^**^+^.


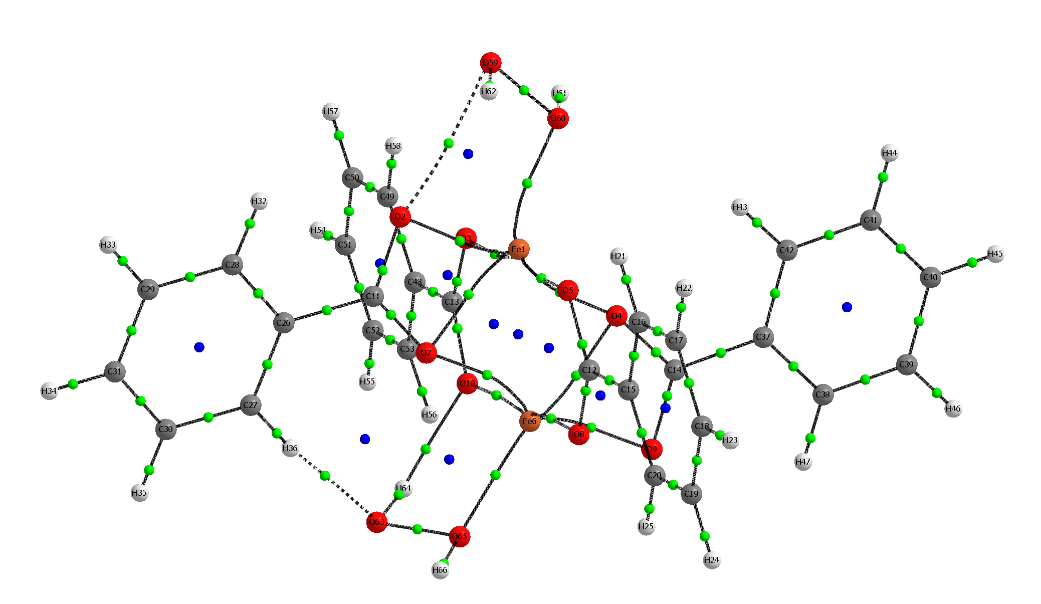


**Fe-BTC-H_2_O_2_**

**
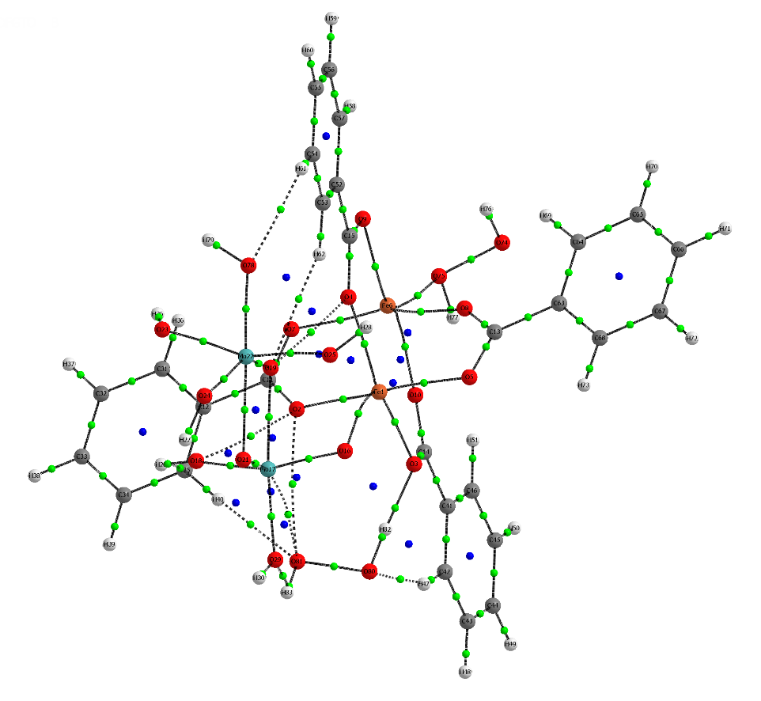
**

**Fe-BTC-PMA-H_2_O_2_**

**Figure S10.** QTAIM graphs of Fe-BTC-H_2_O_2_, Fe-BTC-PMA-H_2_O_2_ catalyst models MOF catalyst models, obtained by the analysis of PBE0/6-31G* electron density functions. Bond Critical Points: Green circles; Ring Critical Points: Blue circles; Bond Paths: Gray solid and dashed lines.

**Table S1:** BET surface area, average pore volume and diameter

| Catalyst | S_BET_ (m^2^g^-1^) | Pore volume  (cm^3^g^-1^) | Pore diameter (nm) |
| --- | --- | --- | --- |
| Fe-BTC | 1,074 | 0.6326 | 2.2 |
| Fe-BTC-PMA | 845 | 0.1055 | 3.7 |

**Table S2:** Surface percentage (from XPS) of different oxygen species

| Catalyst | Oxygen vacancy (%) | Lattice oxygen (%) | -OH group (%) |
| --- | --- | --- | --- |
| Fe-BTC | 39.8 | 42.8 | 17.4 |
| Fe-BTC-PMA | 48.1 | 40.7 | 11.2 |
| PMA | - | 46.1 | 14.4 |
| 3D-Fe-BTC-PMA | 45.3 | 44.6 | 10.1 |

**Table S3:** Surface percentage of different Fe species

| Catalyst | Fe^+2^ (relative %) | Fe^+3^ (relative %) |
| --- | --- | --- |
| Fe-BTC | 58.2 | 41.8 |
| Fe-BTC-PMA | 63.6 | 36.4 |
| 3D-Fe-BTC-PMA | 62.8 | 37.2 |

**Table S4**. Results of non-linear least-squares curve fittings for Fe K-edge EXAFS spectra for Fe-BTC and Fe-BTC-PMA. The curve fitting analysis was performed for the range of 0.363−*R*−2.238 Å, and 2.578−*k*−10.61 Å^−1^,^a^ 0.363−*R*−2.205 Å, and 2.605−*k*−10.66 Å^−1^,^b^

| Sample | Bond | CN | R (Å) | σ^2^  (10^-3^ x Å^2^) | ΔE (eV) |
| --- | --- | --- | --- | --- | --- |
| Fe-BTC^a^ | Fe-O | 6.0 | 2.01 | 7.6 | 2.03 |
| Fe-BTC-PMA^b^ | Fe-O | 8.2 | 2.04 | 6.5 | 2.21 |

**Table S5** Comparison of the apparent Michaelis–Menten constant (*Km*) and the maximum reaction rate (*Vm*) obtained from the double reciprocal plots

| Catalyst | *Km* (Mm) | | *Vm* (M s^−1^) | | Reference |
| --- | --- | --- | --- | --- | --- |
|  | **TMB** | **H_2_O_2_** | **TMB** | **H_2_O_2_** |  |
| CO-MOF | 0.72 | 0.20 | 3.12×10^−7^ | 4.23×10^−7^ | (*6*) |
| CuO@g-C3N4 Cu-MOF | 0.16 | 0.25 | 1.67×10^−8^ | 0.67×10^−8^ | (*7*) |
| S, N-CD@Ce-MOF | 1.145 | 0.828 | 5.17×10^−8^ | 2.50×10^−8^ | (*8*) |
| NH2-CuBDC | 14.52 | 3.2 | 13.2×10^−8^ | 1.33×10^−8^ | (*9*) |
| ZrFe-MOF | 4.91 | 16.79 | 6.93×10^−8^ | 9.43×10^−8^ | (*10*) |
| HRP | 0.434 | 3.7 | 10 ×10^−8^ | 8.71×10^−8^ | (*11*) |
| Fe-BTC-PMA | 0.126 | 36.5 | 7.4×10^−8^ | 31.7× 10^−8^ | Our work |
| Fe-BTC | 0.08 | 18.5 | 3.1×10^−8^ | 11.2× 10^−8^ | Our work |
| PMA | 0.06 | 3.5 | 1.5×10^−8^ | 0.45× 10^−8^ | Our work |

**Table S6.** QTAIM properties of some selected key bond critical points (BCPs) in Fe-BTC-H_2_O_2_, Fe-BTC-PMA-H_2_O_2_ MOF catalyst models, calculated via wave function analysis at PBE0/6-31G* level of theory.

|  | $\boldsymbol{\rho}_{\boldsymbol{b}}$ | $\boldsymbol{\nabla}^{\boldsymbol{2}}\boldsymbol{\rho}_{\boldsymbol{b}}$ | $\mathbf{V}_{\mathbf{b}}$ | $\mathbf{G}_{\mathbf{b}}$ | $\mathbf{H}_{\mathbf{b}}$ | ${\mathbf{\vert}\mathbf{V}_{\mathbf{b}}\mathbf{\vert}}/{\mathbf{G}_{\mathbf{b}}}$ |
| --- | --- | --- | --- | --- | --- | --- |
| **Fe-BTC-H_2_O_2_** |  |  |  |  |  |  |
| **BCPs** |  |  |  |  |  |  |
| **Fe6-O65(H_2_O_2_)** | 0.0586 | 0.4214 | 0.1037 | -0.1021 | 0.0016 | 0.9845 |
| **O10-H64(H_2_O_2_)** | 0.0322 | 0.1149 | 0.0288 | -0.0289 | -0.0001 | 1.0034 |
| **H36-O63(H_2_O_2_)** | 0.0109 | 0.0370 | 0.0087 | -0.0082 | 0.0005 | 0.9425 |
| **Fe1-O60(H_2_O_2_)** | 0.0602 | 0.3612 | 0.0924 | -0.0945 | -0.0021 | 1.0227 |
| **O2-O59(H_2_O_2_)** | 0.0145 | 0.0600 | 0.0137 | -0.0124 | 0.0013 | 0.9051 |
|  |  |  |  |  |  |  |
| **RCPs** |  |  |  |  |  |  |
| **Fe1-O60-O59-O2** | 0.0136 | 0.0562 | 0.0134 | -0.0127 | 0.0007 | 0.9477 |
| **Fe6-O65-O63-H64-O10** | 0.0160 | 0.0760 | 0.0177 | -0.0166 | 0.0011 | 0.9378 |
| **Fe-BTC-PMA-H_2_O_2_** |  |  |  |  |  |  |
| **BCPs** |  |  |  |  |  |  |
| **Fe6-O75(H_2_O_2_)** | 0.0600 | 0.4219 | 0.1049 | -0.1037 | 0.0012 | 0.9885 |
| **Mo17-O81(H_2_O_2_)** | 0.0196 | 0.0537 | 0.0147 | -0.0160 | -0.0013 | 1.0884 |
| **O3-H82(H_2_O_2_)** | 0.0472 | 0.1647 | 0.0421 | -0.0430 | -0.0009 | 1.0213 |
| **O29-H83(H_2_O_2_)** | 0.0295 | 0.1193 | 0.0289 | -0.0279 | 0.0010 | 0.9653 |
|  |  |  |  |  |  |  |
| **RCPs** |  |  |  |  |  |  |
| **Mo17-O81-O80-H82-O3-Fe1-O16** | 0.0072 | 0.0342 | 0.0071 | -0.0057 | 0.0014 | 0.8028 |
| **Mo17-O81-H83-O29** | 0.0194 | 0.0551 | 0.0149 | -0.0162 | -0.0013 | 1.0872 |

**Table S7:** The cartesian coordinates of atoms in the optimized structure of Fe-BTC, Fe-BTC-PMA, Fe-BTC-H_2_O_2_, Fe-BTC-PMA-H_2_O_2_ catalyst models, calculated at PBE0/6-31G* level of theory.

**Fe-BTC**

**Atom Cartesian coordinates (XYZ)**

Fe -0.56660 -0.10460 -0.34830

O 0.47960 -1.75240 -0.88850

O -2.07840 -1.19460 0.32340

O -1.42790 1.47910 0.42130

O 0.49720 1.08590 -1.55990

Fe -2.20210 0.53280 -2.39380

O -1.07680 -1.03430 -2.23260

O -0.75630 1.37420 -3.39520

O -2.44960 2.16580 -1.40360

O -3.47030 -0.41620 -1.25380

C -0.16100 -1.90150 -1.95510

C 0.31760 1.53250 -2.73190

C -3.22100 -1.10080 -0.20960

C -2.10820 2.34610 -0.19590

C 1.42400 2.27170 -3.37620

C 2.62490 2.43610 -2.70700

C 3.65990 3.12630 -3.30800

C 3.49140 3.65320 -4.57800

C 2.28930 3.49030 -5.24710

C 1.25550 2.79910 -4.64540

H 2.73160 2.02150 -1.72720

H 4.59050 3.25400 -2.79240

H 4.29410 4.18950 -5.04470

H 2.16120 3.90000 -6.22900

H 0.31830 2.66090 -5.14090

C 0.11040 -3.00590 -2.87490

C -0.59820 -3.13100 -4.05790

C 1.09140 -3.92720 -2.54410

C 1.36400 -4.97590 -3.39940

C -0.32520 -4.17960 -4.91400

C 0.65540 -5.10080 -4.58350

H 1.62340 -3.80660 -1.62390

H 2.12130 -5.69040 -3.14790

H 0.86700 -5.91490 -5.24820

H -0.87000 -4.28000 -5.83080

H -1.35110 -2.41040 -4.29770

C -2.51200 3.58360 0.49170

C -3.24800 4.54060 -0.18680

C -3.62590 5.70140 0.45960

C -3.26730 5.90160 1.78280

C -2.53120 4.94350 2.46090

C -2.15260 3.78320 1.81410

H -1.58550 3.02840 2.31680

H -2.25650 5.10180 3.48430

H -3.56090 6.80250 2.28450

H -4.19440 6.44520 -0.06140

H -3.51130 4.36400 -1.20820

C -4.34220 -1.83900 0.41220

C -4.11490 -2.60790 1.54120

C -5.15870 -3.29950 2.12510

C -6.42960 -3.21970 1.57990

C -6.65750 -2.44880 0.45160

C -5.61270 -1.75850 -0.13210

H -7.24010 -3.75560 2.03330

H -7.64210 -2.38710 0.03300

H -5.76590 -1.15520 -1.00130

H -4.98520 -3.89580 2.99840

H -3.12530 -2.65250 1.94360

**Fe-BTC-PMA**

**Atom Cartesian coordinates (XYZ)**

Fe -7.06760 -1.29000 -1.81390

O -8.06130 -0.18080 -0.43480

O -5.35980 -0.84650 -0.82580

O -7.65900 -2.99570 -0.79960

O -6.04560 -2.54970 -3.00800

Fe -5.93020 -2.49820 0.21060

O -7.06250 -1.23770 1.26430

O -5.00180 -3.49790 -1.27260

O -6.96420 -4.15910 0.91260

O -4.12530 -1.57120 0.81570

C -7.80100 -0.33340 0.81720

C -8.40990 0.61300 1.78160

C -5.17790 -3.36860 -2.50040

C -4.29390 -0.73170 -0.09660

C -7.82020 -3.99570 0.00960

O -6.75510 0.53870 -3.01370

Mo -8.32900 1.00390 -3.47790

O -9.49590 1.16080 -2.06180

O -8.69570 -1.06840 -3.02690

H -9.20720 0.63060 -1.25190

O -8.83780 0.26780 -4.99990

Mo -9.40770 -1.84110 -4.60710

O -10.99740 -1.11570 -3.88150

O -9.82430 -1.55390 -6.37220

O -7.75350 -2.50580 -4.77650

H -11.10260 -0.67340 -3.02910

H -9.72540 -0.71650 -6.84750

H -6.93950 -2.62180 -4.14160

O -8.16210 2.75700 -3.91790

H -8.07030 3.70390 -4.02400

C -8.25930 0.36780 3.13730

C -8.80860 1.23390 4.06200

C -9.50090 2.35370 3.63210

C -9.64190 2.60720 2.27810

C -9.09730 1.73720 1.35290

H -7.71340 -0.49850 3.44540

H -8.69580 1.04040 5.10970

H -9.92580 3.02820 4.34900

H -10.17150 3.47730 1.94570

H -9.19880 1.94020 0.30860

C -3.35380 0.35650 -0.37150

C -3.60060 1.24080 -1.40920

C -2.70180 2.26000 -1.66150

C -1.56610 2.39050 -0.87860

C -1.32310 1.50410 0.15860

C -2.21890 0.48420 0.41180

H -4.48480 1.11980 -2.00070

H -2.88230 2.94690 -2.46380

H -0.86990 3.18150 -1.07710

H -0.44310 1.60870 0.76060

H -2.05690 -0.21640 1.20450

C -8.96130 -4.90300 -0.14070

C -9.78770 -4.82810 -1.24780

C -10.84660 -5.70870 -1.36850

C -11.08130 -6.65160 -0.38200

C -10.25040 -6.72610 0.72440

C -9.18520 -5.85580 0.84080

H -8.52080 -5.89830 1.67820

H -10.43000 -7.46000 1.48400

H -11.90500 -7.33130 -0.47790

H -11.48130 -5.66910 -2.23120

H -9.59000 -4.11120 -2.01400

C -4.37170 -4.18530 -3.43180

C -3.49760 -5.12420 -2.90660

C -2.72850 -5.89910 -3.75160

C -2.82970 -5.73220 -5.12300

C -3.69840 -4.79070 -5.64920

C -4.46920 -4.01610 -4.80370

H -3.43500 -5.23230 -1.84450

H -2.05480 -6.62680 -3.34620

H -2.23260 -6.33310 -5.78010

H -3.77370 -4.66100 -6.70990

H -5.13790 -3.28590 -5.20700

O -10.16100 -3.51420 -4.31290

H -11.10190 -3.66130 -4.14280

**Fe-BTC-H2O2**

**Atom Cartesian coordinates (XYZ)**

Fe -1.50070 -2.14780 -0.11700

O -1.50420 -4.19310 -0.13800

O -1.72470 -2.11890 1.87680

O -2.08440 -0.23660 -0.04330

O -1.73250 -2.07630 -2.08620

Fe -3.96180 -1.10540 -0.09040

O -3.38350 -3.08070 -0.08540

O -3.78720 -1.17620 -2.06200

O -3.88600 0.98230 -0.04370

O -3.83780 -1.36170 1.90590

C -2.77100 -4.20460 -0.09250

C -2.75740 -1.60960 -2.66440

C -2.75900 -1.74160 2.48580

C -2.63580 0.93150 -0.08470

C -2.75800 -1.57390 -4.14610

C -1.66970 -2.06500 -4.84710

C -1.66850 -2.03240 -6.22860

C -2.75530 -1.50570 -6.90730

C -3.84250 -1.01090 -6.20580

C -3.84310 -1.04550 -4.82430

H -0.84130 -2.46300 -4.30020

H -0.82780 -2.41240 -6.77370

H -2.75400 -1.47910 -7.97930

H -4.68020 -0.60020 -6.73300

H -4.66820 -0.66510 -4.26050

C -3.52200 -5.45770 -0.03210

C -4.87790 -5.43950 0.25270

C -2.86350 -6.65780 -0.24630

C -3.56630 -7.84470 -0.19520

C -5.57750 -6.62930 0.31380

C -4.92350 -7.82870 0.08410

H -1.81430 -6.64530 -0.45500

H -3.06310 -8.77430 -0.36850

H -5.46950 -8.75020 0.12740

H -6.62400 -6.62320 0.54230

H -5.36220 -4.50670 0.45190

C -1.79680 2.12840 -0.16710

C -2.39470 3.37710 -0.12950

C -1.61480 4.51400 -0.20790

C -0.23960 4.39820 -0.33040

C 0.35720 3.14850 -0.37550

C -0.42270 2.01140 -0.29270

H 0.01750 1.03700 -0.33640

H 1.42020 3.06400 -0.47830

H 0.36640 5.28030 -0.39420

H -2.07260 5.48210 -0.17690

H -3.45940 3.43680 -0.04010

C -2.74980 -1.74640 3.96560

C -1.67190 -2.29080 4.64450

C -1.66000 -2.29990 6.02550

C -2.72430 -1.75750 6.72750

C -3.79970 -1.20790 6.04970

C -3.81340 -1.20440 4.66820

H -2.71460 -1.76210 7.79950

H -4.61920 -0.78440 6.59460

H -4.63150 -0.77600 4.12840

H -0.82970 -2.72530 6.55240

H -0.86150 -2.70530 4.08330

O 1.08340 -3.43330 0.07200

O 0.53830 -2.06300 0.13610

H 0.83240 -1.75700 1.00710

H 0.73320 -3.72650 -0.80210

O -6.16300 -2.39560 1.26690

H -5.62130 -1.85020 1.89880

O -5.93760 -1.57080 0.06370

H -6.44540 -2.02690 -0.62140

**Fe-BTC-PMA-H_2_O_2_**

**Atom Cartesian coordinates (XYZ)**

Fe 0.04780 -2.15680 0.83500

O -0.82730 -1.89430 -0.92320

O -0.70010 -0.31680 1.16310

O -0.39310 -4.10900 0.60850

O 0.67050 -2.26500 2.71940

Fe -2.58230 -3.14610 1.20700

O -2.75780 -3.01130 -0.80800

O -1.37130 -3.11670 2.79330

O -2.27840 -5.12020 1.11280

O -2.74800 -1.14770 1.35690

C -1.91010 -2.31080 -1.42940

C -2.17710 -1.94720 -2.84210

C -0.26920 -2.82120 3.35440

C -1.95030 -0.16640 1.33440

C -1.04300 -5.17470 0.85430

O 1.85060 -1.59900 0.01470

Mo 2.54200 -1.73870 -1.53550

O 1.50230 -1.91150 -2.98590

O 2.45640 -3.79020 -1.04930

H 0.91540 -1.81580 -3.72990

O 4.15210 -2.49920 -1.88390

Mo 4.04450 -4.57530 -1.38930

O 3.22060 -5.24300 -2.97600

O 5.64570 -4.39390 -2.27960

O 4.67590 -4.44180 0.31650

H 2.25960 -5.30780 -3.06500

H 5.86650 -3.63730 -2.83510

H 4.79600 -4.89190 1.15810

O 3.49260 -0.12080 -1.72610

H 4.39330 -0.07180 -2.06900

C -3.15010 -2.61780 -3.55170

C -3.37280 -2.29900 -4.88030

C -2.63730 -1.29550 -5.48680

C -1.68120 -0.60370 -4.76410

C -1.44630 -0.92970 -3.44110

H -3.71130 -3.38370 -3.05890

H -4.11670 -2.82610 -5.43650

H -2.81430 -1.04380 -6.51390

H -1.13510 0.19640 -5.22660

H -0.72460 -0.39630 -2.85860

C -2.49520 1.19860 1.52570

C -1.72710 2.31680 1.23940

C -2.25370 3.58000 1.43710

C -3.53990 3.72940 1.92450

C -4.31220 2.61290 2.20080

C -3.79100 1.35190 1.99290

H -0.74720 2.20400 0.83040

H -1.66220 4.44350 1.20810

H -3.94290 4.71050 2.08240

H -5.31080 2.72800 2.57150

H -4.37930 0.48310 2.19380

C -0.35890 -6.47730 0.86850

C 0.99510 -6.55090 0.59950

C 1.63920 -7.77530 0.64680

C 0.92350 -8.92050 0.95720

C -0.43650 -8.84710 1.21310

C -1.07720 -7.62420 1.16770

H -2.12410 -7.53770 1.35840

H -0.98740 -9.73590 1.44440

H 1.42710 -9.86720 0.99400

H 2.68770 -7.82250 0.43300

H 1.51980 -5.65200 0.34380

C -0.05960 -3.20170 4.76480

C -0.86780 -4.15660 5.35620

C -0.64320 -4.53540 6.66550

C 0.37930 -3.93970 7.38940

C 1.18170 -2.97490 6.80150

C 0.97080 -2.61680 5.48440

H -1.65380 -4.60400 4.78300

H -1.25920 -5.28840 7.11990

H 0.54850 -4.22710 8.40820

H 1.96740 -2.51220 7.36470

H 1.58660 -1.88960 5.00380

O -4.25390 -3.49030 3.63570

O -4.33700 -3.04020 2.23930

H -3.83890 -4.36170 3.49210

H -4.50100 -2.08490 2.32740

O 4.04260 -6.43700 -0.94430

H 3.78990 -7.05450 -1.64250

O 1.08760 1.19740 -0.06880

O 1.10110 0.68580 -1.45010

H 0.47550 0.56890 0.38510

H 1.96470 1.03450 -1.73340

*References*

1. V. V. Karve *et al.*, Hybridization of synthetic humins with a metal–organic framework for precious metal recovery and reuse. *ACS Applied Materials & Interfaces* **13**, 60027-60034 (2021).

2. C. Adamo, V. Barone, Toward reliable density functional methods without adjustable parameters: The PBE0 model. *The Journal of chemical physics* **110**, 6158-6170 (1999).

3. F. Neese, The ORCA program system. *Wiley Interdisciplinary Reviews: Computational Molecular Science* **2**, 73-78 (2012).

4. F. Neese, Software update: The ORCA program system—Version 5.0. *Wiley Interdisciplinary Reviews: Computational Molecular Science* **12**, e1606 (2022).

5. R. Bader, AIM2000 Program, v. 2.0. *McMaster University, Hamilton, Canada*, (2000).

6. Z. Deng *et al.*, Cobalt-Based Metal-Organic Framework Nanoparticles with Peroxidase-like Catalytic Activity for Sensitive Colorimetric Detection of Phosphate. *Catalysts* **12**, 679 (2022).

7. M. Lu *et al.*, Cu-MOF derived CuO@g-C3N4 nanozyme for cascade catalytic colorimetric sensing. *Analytical and Bioanalytical Chemistry* **415**, 5949-5960 (2023).

8. R. Jesuraj, P. Perumal, A highly effective peroxidase-mimic nanozyme of S, N-carbon dot-decorated cerium organic framework-based colorimetric detection of Hg2+ ion and thiophanate methyl. *Analytical Methods* **16**, 3562-3576 (2024).

9. S. Liu *et al.*, A smartphone-based fluorescent biosensor with metal-organic framework biocomposites and cotton swabs for the rapid determination of tetrodotoxin in seafood. *Analytica Chimica Acta* **1311**, 342738 (2024).

10. S. Liu *et al.*, Aptamer-controlled reversible colorimetric assay: High-activity bimetallic organic frameworks for the efficient sensing of marine biotoxins. *Chemical Engineering Journal* **469**, 144027 (2023).

11. Q. Liu *et al.*, One-pot synthesis of porphyrin functionalized γ-Fe2O3 nanocomposites as peroxidase mimics for H2O2 and glucose detection. *Materials Science and Engineering: C* **55**, 193-200 (2015).
